# Supplementary material for: Cell-type-specific propagation of visual flicker
Source: Cell Rep. Author manuscript; Available in PMC 2024 Dec 16. (PMC7617239; doi:10.1016/j.celrep.2023.112492)
Supplement: Supplementary Materials [file EMS200285-supplement-Supplementary_Materials.zip › 1-s2.0-S221112472300503X-mmc1.pdf]

Cell Reports, Volume *42*

Supplemental information

Cell-type-specific propagation of visual flicker

Marius Schneider, Athanasia Tzanou, Cem Uran, and Martin Vinck

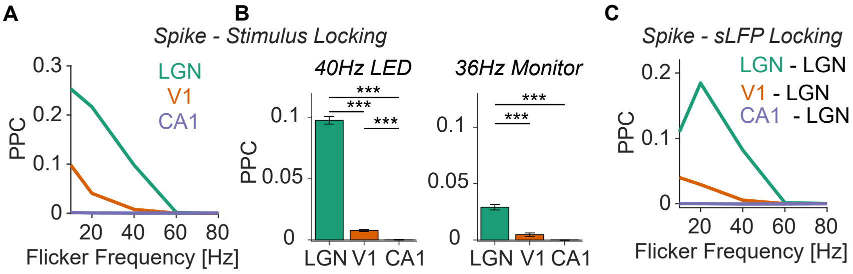

Figure S1: Same as Figure 2 but including only visually responsive neurons. (A) Spike-stimulus phase locking of visually responsive neurons in LGN (n=1332), V1 (n=1028), and CA1 (n=72) during LED flicker stimulation. **(B)** Phase locking to stimulus during 40 Hz LED (left, n_{LGN} = 1332, n_{V1} = 1028, n_{CA1} = 72) and 36 Hz monitor (right, n_{LGN} = 461, n_{V1} = 336, n_{CA1} = 16) flicker presentation. ***P<0.001; **P<0.01; *P<0.05, non-parametric permutation tests, based on 1000 randomizations. **(C)** Spike-sLFP phase locking for different flicker frequencies (presented using a LED) and combinations of spikes and sLFPs: spikes in LGN (n=1332) to sLFPs in LGN, spikes in V1 (n=1028) to sLFP in LGN, and spikes in CA1 (n=72) to sLFP in LGN. Error bars and shadings indicate SEMs.

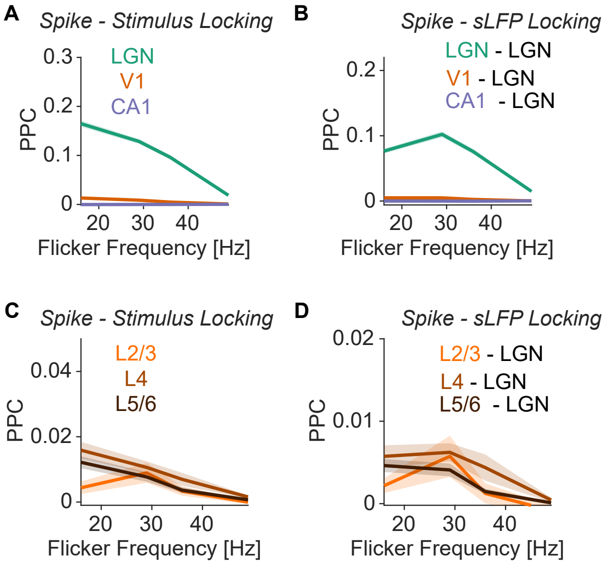

Figure S2: Same as Figure 2 but for monitor stimulation. (A) Spike-stimulus phase locking of neurons in LGN (n=1154), V1 (n=815), and CA1 (n=125) during monitor flicker stimulation. **(B)** Spike-sLFP phase locking for different flicker frequencies (presented using a monitor) and combinations of spikes and sLFPs: spikes in LGN to sLFPs in LGN, spikes in V1 to sLFP in LGN, and spikes in CA1 to sLFP in LGN. Same n as in (A). **(C)** Spike-stimulus phase locking of neurons in different layers of V1 during monitor flicker stimulation ($n_{sup.}$ = 32, $n_{gra.}$ = 279, $n_{inf.}$=504) . **(D)** Spike-sLFP phase locking during monitor flicker presentation between spikes in different V1 layers and the LGN-sLFP. Same n as in (C). Error bars and shadings indicate SEMs.

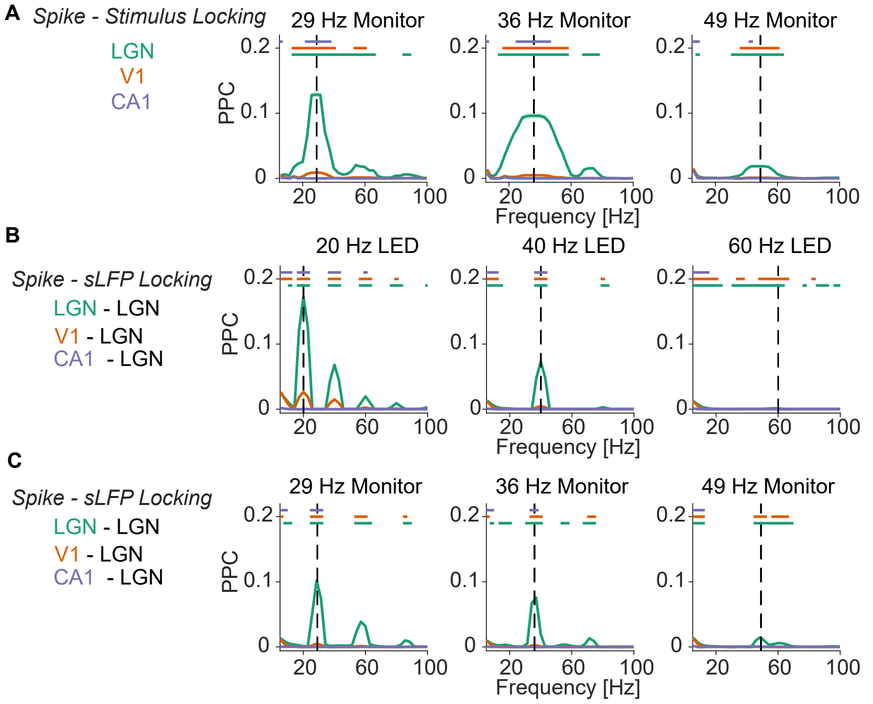

Figure S3: PPC spectra of Neurons in LGN, V1 and CA1, related to Figure 2 and S2. (A) Spike-field phase locking during LED flicker stimulation (measured with PPC) for different combinations of spikes and sLFPs: spikes in LGN (n=2386) to sLFPs in LGN (green); spikes in V1 (n=2091) to sLFP in LGN (orange); spikes in CA1 (n=636) to sLFP in LGN (blue). **(B)** Spike-field phase locking during monitor flicker stimulation (measured with PPC) for different combinations of spikes and sLFPs: spikes in LGN (n=1153 to sLFPs in LGN (green); spikes in V1 (n=815) to sLFP in LGN (orange); spikes in CA1 (n=125) to sLFP in LGN (blue). **(C)** Spike-stimulus phase locking during monitor flicker stimulation (measured with PPC) of neurons in LGN (n=1153, green); V1 (n=815, orange) and CA1 (n=125, blue). Randomization test between cells in different areas, FDR correction for multiple comparisons with a threshold of P<0.05, based on 1000 randomizations. Green line: LGN-V1, orange line: LGN-CA1, blue line: V1-CA1. Shadings indicate SEMs.

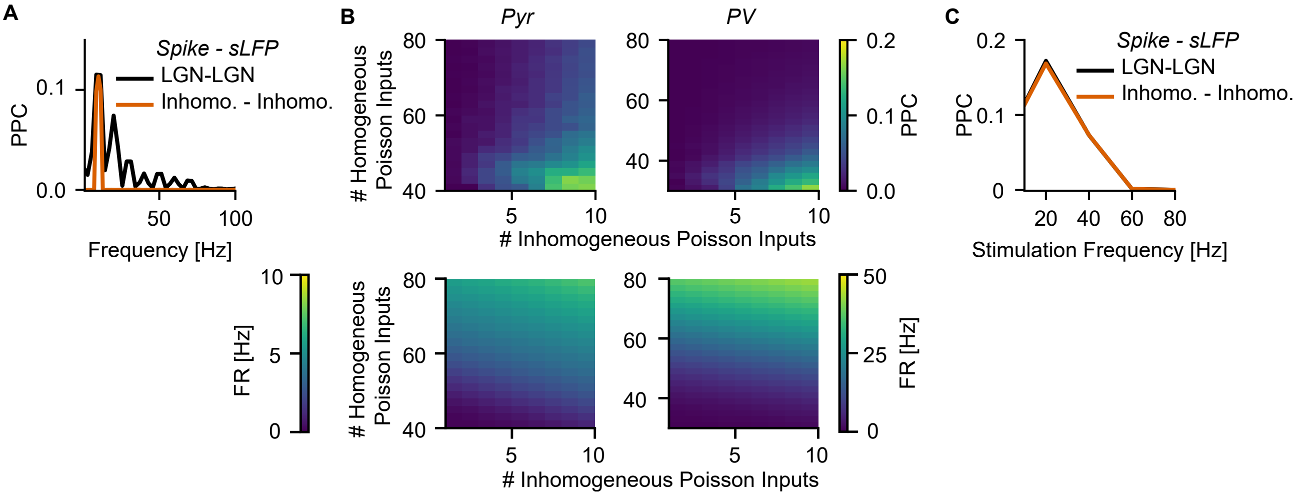

Figure S4: V1 multi-compartmental model during synaptic stimulation, related to Figure 5. (A) Spike-sLFP phase locking of LGN neurons during 10 Hz LED flicker stimulation to LGN population activity (black). Phase locking of inhomogeneous Poisson spiking input neurons (orange). Modulation strength of inhomogeneous Poisson spiking input neurons adjusted to reproduce experimentally observed phase locking in LGN. **(B)** Grid scan of the number of homogeneous and inhomogeneous Poisson spiking input spike trains during 10 Hz stimulation. The top plots show the phase locking (PPC) of the pyramidal (left) and PV+ (right) multi-compartmental model to the inhomogeneous Poisson spiking input population. The bottom plots show the firing rates of the corresponding neuron models. The number of homogeneous and inhomogeneous Poisson spiking input trains was optimized to fit the experimental results of BW and NW spiking neurons during 10 Hz LED stimulation. **(C)** Spike-sLFP phase locking (PPC) of single units in LGN to LGN population activity during stimulation at different frequencies (black). The modulation strength of inhomogeneous Poisson spiking input spike trains was adjusted to match experimental observations.

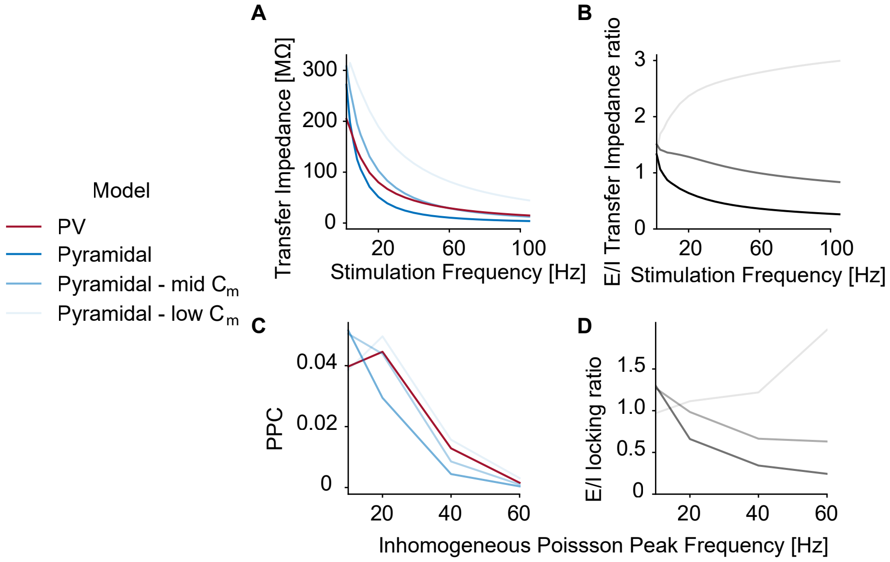

Figure S5: Effects of varying membrane capacitance on dendritic low-pass filtering, related to Figure 5. **(A)** Same simulations as in Figure 5A-D. Transfer impedance of PV+ and pyramidal cell model during sinusoidal current stimulation between 1 and 100 Hz. Increasing the membrane capacitance in pyramidal cell dendrites resulted in a systematic increase in transfer impedance. **(B)** Ratio between the transfer impedance of the pyramidal cell model with scaled membrane capacitance and the PV+ neuron model. **(C)** Same simulations as in Figure 5H-5K. Phase locking of PV+ and pyramidal cell model with scaled membrane capacitance during synaptic stimulation with homogeneous and inhomogeneous spike trains. **(D)** Ratio between phase locking of the pyramidal cell model with scaled dendritic membrane capacitance and the PV+ cell model.
