# Supplementary material for: Cell-type-specific propagation of visual flicker
Source: Cell Rep. Author manuscript; Available in PMC 2024 Dec 16. (PMC7617239; doi:10.1016/j.celrep.2023.112492)
Supplement: Supplementary Materials [file EMS200285-supplement-Supplementary_Materials.zip › 1-s2.0-S221112472300503X-mmc2.pdf]

Cell Reports

Article

Cell-type-specific propagation of visual flicker

Graphical abstract

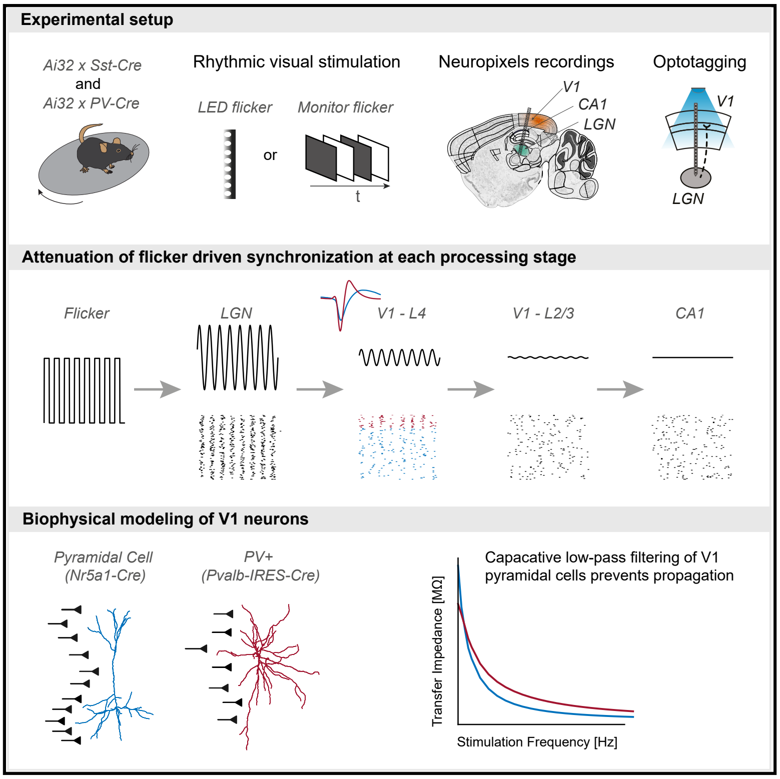

Highlights

- Cell-type-specific study of flicker-induced synchrony in the thalamus, V1, and CA1
- In V1, gamma-frequency flicker preferentially activates fast-spiking interneurons
- In V1, gamma frequencies show little propagation from input layer 4 to layer 2/3
- CA1 neurons are not entrained by visual flicker stimuli at gamma frequencies

Authors

Marius Schneider, Athanasia Tzanou, Cem Uran, Martin Vinck

Correspondence

marius.schneider@esi-frankfurt.de (M.S.),
martin.vinck@esi-frankfurt.de (M.V.)

In brief

Schneider et al. investigate how visual flicker synchronizes neural activity in the thalamus, V1, and hippocampus. High-frequency synchrony showed rapid attenuation with minimal propagation beyond L4 of V1 and no effect on the hippocampus. Visual flicker mainly entrained fast-spiking interneurons in area V1, which was explained by biophysical compartmental models.

Schneider et al., 2023, Cell Reports *42*, 112492
May 30, 2023 © 2023 The Authors.
https://doi.org/10.1016/j.celrep.2023.112492

CellPress

Cell Reports

CellPress
OPEN ACCESS

Article

Cell-type-specific propagation of visual flicker

Marius Schneider,[1,2,*] Athanasia Tzanou,[1] Cem Uran,[1,2] and Martin Vinck[1,2,3,*]
[1]Ernst Strüngmann Institute (ESI) for Neuroscience in Cooperation with Max Planck Society, 60528 Frankfurt am Main, Germany
[2]Donders Centre for Neuroscience, Department of Neuroinformatics, Radboud University Nijmegen, 6525 Nijmegen, the Netherlands
[3]Lead contact
*Correspondence: marius.schneider@esi-frankfurt.de (M.S.), martin.vinck@esi-frankfurt.de (M.V.)
https://doi.org/10.1016/j.celrep.2023.112492

SUMMARY

Rhythmic flicker stimulation has gained interest as a treatment for neurodegenerative diseases and as a method for frequency tagging neural activity. Yet, little is known about the way in which flicker-induced synchronization propagates across cortical levels and impacts different cell types. Here, we use Neuropixels to record from the lateral geniculate nucleus (LGN), the primary visual cortex (V1), and CA1 in mice while presenting visual flicker stimuli. LGN neurons show strong phase locking up to 40 Hz, whereas phase locking is substantially weaker in V1 and is absent in CA1. Laminar analyses reveal an attenuation of phase locking at 40 Hz for each processing stage. Gamma-rhythmic flicker predominantly entrains fast-spiking interneurons. Optotagging experiments show that these neurons correspond to either parvalbumin (PV+) or narrow-waveform somatostatin (Sst+) neurons. A computational model can explain the observed differences based on the neurons' capacitative low-pass filtering properties. In summary, the propagation of synchronized activity and its effect on distinct cell types strongly depend on its frequency.

INTRODUCTION

Rhythmic flicker stimulation has gained increased interest in the context of therapeutic methods for neurodegenerative diseases. For instance, recent studies have shown that 40 Hz visual flicker stimulation can reduce beta-amyloid plaques in Alzheimer's disease mouse models.[1,2] It has been suggested that these effects of high-frequency rhythmic stimulation depend on brain-wide entrainment including the hippocampus and medial prefrontal cortex (mPFC).[3] Furthermore, rhythmic visual stimulation is a well-established method to study the processing of visual information[4] and has been used as a technique for human magnetoencephalography (MEG) and electroencephalography (EEG) recordings to track neural processing across various stages through "frequency tagging."[5–8] Thus, it is important to determine the neural mechanisms through which flicker-induced synchronization propagates across cortical levels and how flicker stimuli influence distinct cell types in the local circuit.

Synchronized activity can result from either rhythmic external inputs or endogenous network interactions. Several theoretical and empirical studies suggest that synchronized activity may facilitate the processing of information by enhancing the impact of spikes on post-synaptic targets.[9–11] Synchronization of neural responses may be especially critical for information flow in the case of sparse feedforward connections, e.g., in the case of thalamocortical communication.[12] Furthermore, functional studies on inter-areal communication suggest that feedforward influences are particularly strong for high-frequency rhythmic activity.[13,14]

Yet, several observations suggest that high-frequency synchronization might not be conductive to signal propagation: first, it is evident that there is perceptual filtering of flicker stimuli above a certain frequency (flicker fusion threshold). One possibility is that flicker-induced synchronization does effectively propagate across various stages of the cortical hierarchy but is perceptually filtered out due to other cortical mechanisms (e.g., in higher processing stages). However, studies of mass population activity in humans (MEG) suggest that high-frequency flicker stimuli may not effectively propagate beyond the primary visual cortex.[15] Second, low-frequency synchronization is typically seen at a much larger spatial scale than local high-frequency synchronization.[16–18] Third, the passive integration properties of single neurons related to the cable equation cause dendritic low-pass filtering, especially in pyramidal neurons.[19–22] However, the combination of active and passive integration properties can also create resonance behavior, i.e., the selective amplification of information in a specific frequency range.[23–25] Thus, it remains overall unclear how synchronization driven by rhythmic stimulation propagates throughout the cortex and how different components of the microcircuit are affected, depending on frequency.

To investigate this, we used Neuropixels to record from multiple processing stages in the mouse brain simultaneously (lateral geniculate nucleus [LGN], different layers of the primary visual cortex [V1], CA1 hippocampus) while presenting (LED and monitor) flicker stimuli at different frequencies. Using optotagging, we distinguished the activity of excitatory neurons and specific GABAergic subtypes, namely parvalbumin (PV+) and somatostatin (Sst+) interneurons. To explain our experimental observations, we performed detailed multicompartmental modeling of mouse V1 cells to investigate the filtering properties of the different cell types.

This is an open access article under the CC BY license (http://creativecommons.org/licenses/by/4.0/).

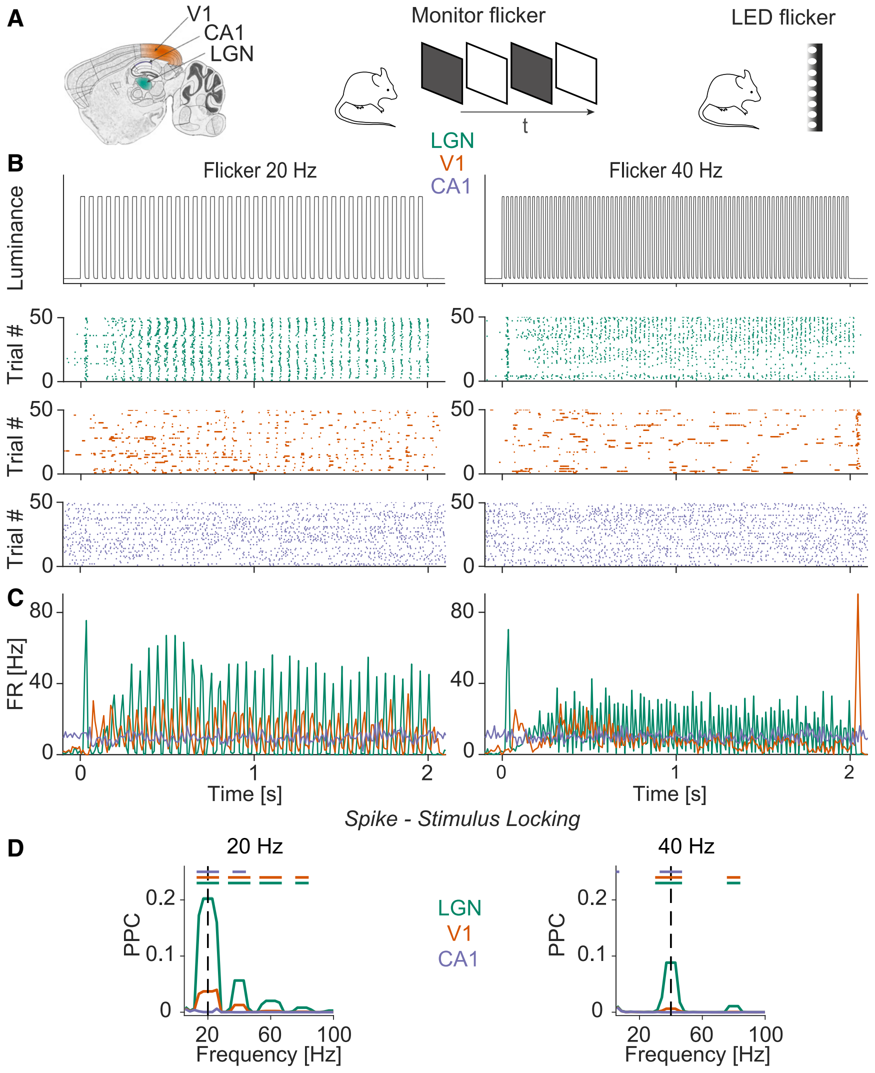

Figure 1. Illustration of the experiment

(A) We made simultaneous Neuropixels recordings from LGN, V1, and CA1 while presenting fast flickering stimuli of different frequencies. Flicker frequencies were presented in randomized order. The rhythmic flicker was presented using either a full-screen monitor or LEDs.

(B) Measured luminance change (using photodiode) of the LED for 20 (top left) and 40 Hz (top right) flicker frequency (y axis has arbitrary units). Raster plot of example neurons in the LGN (green), V1 (orange), and CA1 (blue).

(C) Peristimulus time histogram (PSTH) of neurons shown in (B).

(D) Phase locking of neurons in LGN (n = 2,386), V1 (n = 2,091), and CA1 (n = 636) to the flicker stimulus (randomization test between cells in different areas, false discovery rate [FDR] correction for multiple comparisons with a threshold of $p < 0.05$, based on 1,000 randomizations). Green line: LGN-V1, orange line: LGN-CA1, blue line: V1-CA1. Shadings indicate SEMs.

RESULTS

We recorded isolated single units from areas LGN, V1, and CA1 using Neuropixels probes while mice were placed on a running disk (Figure 1A; see STAR Methods). Visual flicker stimuli (frequencies between 10 and 80 Hz) were presented using either a monitor or an array of LEDs (Figures 1A and 1B). In the case of monitor flicker, we flashed full-field black-and-white stimuli. In the case of the LED arrays, we used similar luminance settings as in previous studies that examined the effect of visual flicker on neurodegeneration.[1] We quantified the locking of individual spikes to the flicker stimuli by first extracting the phase of each spike relative to the flicker cycle and then computing the pairwise phase consistency (PPC) (Figure 1D). The PPC is a measure of phase locking that is unbiased by spike count or firing rates.[26]

Significant phase locking to the flicker stimuli was observed in areas LGN and V1 but not in CA1 (Figures 1B and 1C for example neurons, and Figure 2A for population analysis). For all frequencies, phase locking was stronger in the LGN than in V1 (Figure 2A; see Figure 1D for PPC spectra). Both in V1 and the LGN, phase locking decreased with frequency (Figure 2A). Around 40 Hz, LGN units still exhibited significant phase locking to the flicker stimuli, whereas phase locking was an order of magnitude weaker in area V1 (Figure 2B). At 60 Hz and beyond, we did not observe significant phase locking in any of the areas (Figure 2A). Restricting our analysis to visually responsive neurons did not change our results (Figure S1). Similar phase locking patterns were observed for LED and monitor flicker stimuli (Figures 2B, S2A, and S2B for summary plots of monitor stimuli, and Figure S3A for PPC spectra during monitor stimulation).

To analyze the phase locking of V1 neurons across cortical layers, we identified different layers using current source density (CSD) analysis (see STAR Methods). Neurons in the input layer (L4) showed the strongest phase locking to the flicker stimuli, both for monitor and LED flicker (Figures 2D and S2C). Locking was substantially weaker in layer 2/3 (L2/3), especially for the 40 Hz LED flicker stimuli (Figure 2E).

It is possible that network synchronization may not have occurred exactly at the frequencies of the flicker stimuli. We therefore also quantified the phase locking of single units to the population activity in the LGN. Because of the geometric arrangement of excitatory cells (closed field), the LGN does not necessarily produce an informative and strictly local local field potential (LFP). We therefore constructed a "surrogate LFP" (sLFP) for the LGN by summing all LGN spikes[27,28] (see STAR Methods). Phase locking was then computed as the PPC between spikes and the sLFP. We found that phase locking to the LGN-sLFP showed similar differences between areas and frequencies compared with phase locking to the flicker stimuli

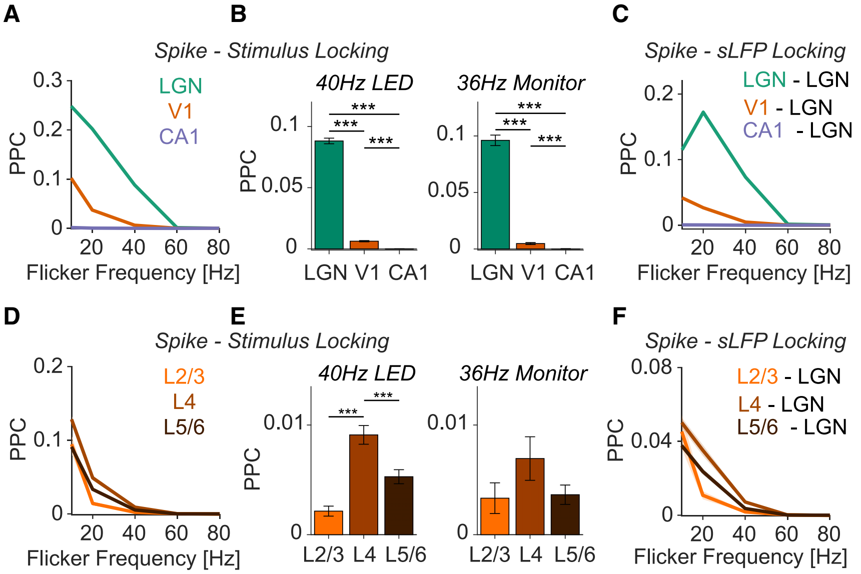

Figure 2. Propagation of flicker-induced synchronization across processing stages

(A) Spike-stimulus phase locking of neurons in LGN (n = 2,386), V1 (n = 2,091), and CA1 (n = 636) during LED flicker stimulation. Phase locking was measured with the unbiased pairwise phase consistency (PPC; see STAR Methods).

(B) Phase locking to the stimulus during 40 Hz LED (left, n_{LGN} = 2,386, n_{V1} = 2,091, n_{CA1} = 636) and 36 Hz monitor (right, n_{LGN} = 1,153, n_{V1} = 815, n_{CA1} = 125) flicker presentation. ***$p < 0.001$; **$p < 0.01$; *$p < 0.05$, non-parametric permutation tests, based on 1,000 randomizations.

(C) Spike-sLFP phase locking for different flicker frequencies (presented using LEDs) and combinations of spikes and sLFPs: spikes in LGN to sLFPs in LGN, spikes in V1 to sLFP in LGN, and spikes in CA1 to sLFP in LGN. The sLFP is the surrogate LFP constructed by summing all LGN spikes together and low-pass filtering (see STAR Methods).

(D) Spike-stimulus phase locking of neurons in different layers of V1 during LED flicker stimulation ($n_{sup.}$ = 80, $n_{gra.}$ = 604, $n_{inf.}$ = 1,407).

(E) PPC between neurons in different V1 layers and the stimulus during 40 Hz LED (left, $n_{sup.}$ = 80, $n_{gra.}$ = 604, $n_{inf.}$ = 1,407) and 36 Hz monitor (left, $n_{sup.}$ = 32, $n_{gra.}$ = 279, $n_{inf.}$ = 504) stimulus presentation. ***$p < 0.001$; **$p < 0.01$; *$p < 0.05$, non-parametric permutation tests, based on 1,000 randomizations.

(F) Spike-sLFP phase locking during LED flicker presentation between spikes in different V1 layers and the LGN-sLFP. Error bars and shadings indicate SEMs.

(Figures 2C and 2F; see Figures S2B and S2D for phase locking during monitor stimulation). Furthermore, the phase locking spectra showed narrow-band peaks at the frequencies of the flicker, showing that synchronization was indeed restricted to the flicker frequency (Figures S3B and S3C).

Together, these analyses indicate that at gamma frequencies, phase locking shows a substantial decrease from LGN to the input layer (L4) of V1 and then further decreases toward L2/3, with no phase locking observed at higher levels of the cortical hierarchy (CA1).

Fast stimuli primarily recruit fast-spiking interneurons

To analyze the responses of distinct V1 cell types to flicker stimuli, we distinguished cell types based on action potential waveforms (Figure 3) and, in a subsequent figure, validated these findings using optotagging (Figure 4). Consistent with previous work, V1 neurons were clearly divided into two categories having broad (BW) and narrow (NW) waveforms (Figure 3A). These categories correspond to putative excitatory neurons and fast-spiking interneurons, respectively (see also Figure 4).[29] To quantify the relative locking strengths of BW and NW neurons, we computed an "E/I" ratio, which was defined as the ratio of PPC values of BW compared with NW neurons (Figure 3C). This analysis revealed that at low frequencies, BW and NW neurons showed approximately similar phase locking values (Figures 3B and 3C). However, for higher frequencies, NW neurons were significantly more phase locked than BW neurons (Figures 3B–3D). We furthermore examined the laminar profile of locking. At gamma frequencies, the strongest phase locking was observed for L4 NW neurons, and phase locking was very weak in L2/3 excitatory neurons (Figure 3D). Similar findings were made for LED and monitor flicker (Figures 3B–3D).

We note that these conclusions did not depend on the specific phase locking analysis method used but were also obtained with a predictive regression model in which spike timing was predicted from the phase of the flicker stimulus (Figure 3E; see STAR Methods). Consistent with the phase locking analysis, the spike timing could be substantially better predicted for NW than for BW neurons (Figure 3F).

Optotagging experiments were performed in Ai32 x PV-Cre and Ai32 x Sst-Cre animals to further distinguish between different GABAergic subtypes (Figures 4A and 4D). Consistent with the analysis of NW neurons, we found that PV+ neurons (which typically had NWs) were more strongly locked to gamma-frequency flicker stimuli than BW neurons (Figures 4B and 4C). For Sst+ neurons, we analyzed NW and BW Sst+ neurons separately. At gamma frequencies, NW Sst+ neurons exhibited substantial phase locking to visual flicker stimuli, whereas BW Sst+ neurons were relatively weakly locked (Figures 4D and 4E).

Filtering properties of V1 principal cells

We wondered if the observed differences between cell types could be explained by their respective biophysical properties. To this end, we used detailed biophysical multicompartmental models from the Allen Institute to test the filtering properties of different V1 cell types. The multicompartmental models contained a set of 10 active membrane conductances placed in the soma and detailed reconstructed morphologies. Model parameters were optimized to reproduce the firing behavior during somatic whole-cell patch clamp recordings in slices (see STAR Methods).[30–32]

We first tested the capacitive filtering effects of dendrites of different cell types during the passive propagation of signals from a dendritic arbor to the soma. To this end, we injected

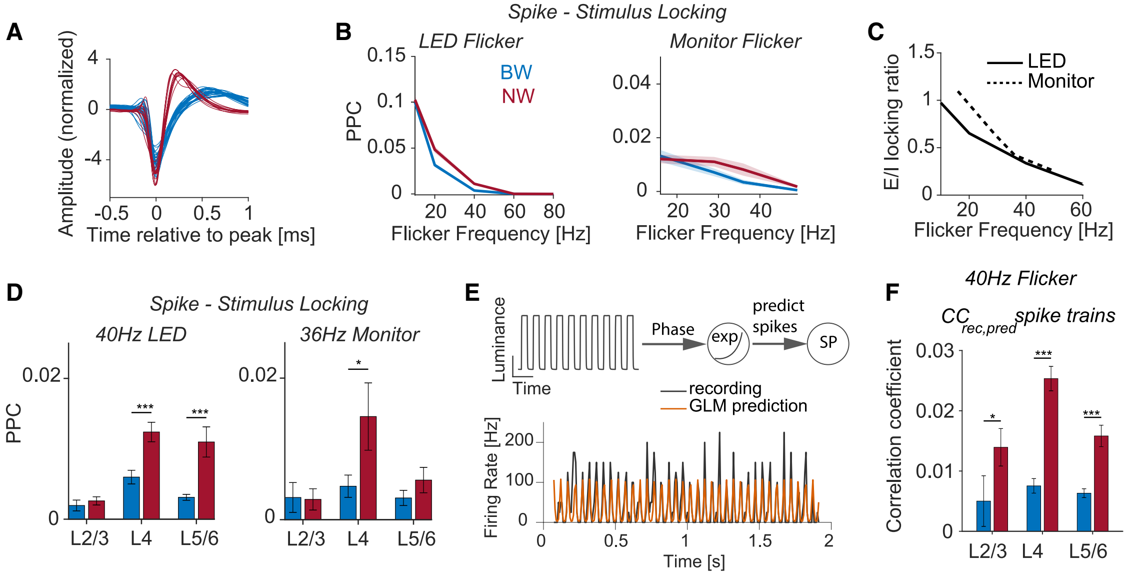

Figure 3. Fast frequencies predominantly drive narrow-waveform interneurons

(A) Normalized spike waveforms of neurons in V1.

(B) Phase locking between different cell classes in V1 and the LED (left, $n_{BW} = 1,316$, $n_{NW} = 664$) and monitor (right, $n_{BW} = 474$, $n_{NW} = 274$) during flicker stimulation at different frequencies.

(C) The ratio between phase locking of excitatory and inhibitory cells to the stimulus using LEDs (black) and monitor (green).

(D) Phase locking of different cell classes in the different layers of V1 to 40 Hz LED (left, broad waveform [BW]: $n_{sup.} = 38$, $n_{gra.} = 300$, $n_{inf.} = 978$; narrow waveform [NW]: $n_{sup.} = 37$, $n_{gra.} = 269$, $n_{inf.} = 358$) and 36 Hz monitor (right, BW: $n_{sup.} = 11$, $n_{gra.} = 182$, $n_{inf.} = 375$; NW: $n_{sup.} = 21$, $n_{gra.} = 104$, $n_{inf.} = 109$) stimulus. ***$p < 0.001$; **$p < 0.01$; *$p < 0.05$, non-parametric permutation tests, based on 1,000 randomizations.

(E) Illustration of statistical model employed to predict spike times of V1 neurons from the instantaneous stimulus phase (top). PSTH of recorded example neuron in V1 during 20 Hz LED flicker stimulation (black) and corresponding prediction from the model (orange).

(F) The correlation coefficient between recorded and predicted spike trains of neurons in different layers of V1 (BW: $n_{sup.} = 31$, $n_{gra.} = 274$, $n_{inf.} = 932$; NW: $n_{sup.} = 36$, $n_{gra.} = 261$, $n_{inf.} = 353$) (*$p < 0.05$; **$p < 0.01$; ***$p < 0.001$; Wilcoxon Mann-Whitney test). Error bars and shadings indicate SEMs.

sinusoidal currents of different frequencies in dendritic compartments 150 μm from the soma in both pyramidal and PV+ neuron models (Figure 5A). The neuron's response was examined via the voltage fluctuations in the soma, and the transfer impedance was computed for each stimulation frequency (Figure 5B).[22] We found that for low frequencies, pyramidal cells had a higher transfer impedance than PV+ neurons. To quantify the relative filtering difference between pyramidal and PV+ neurons, we calculate the transfer impedance ratio, defined as the ratio of the transfer impedance of pyramidal and PV+ neurons (Figures 5D and 5G). With increasing stimulation frequency, the transfer impedance of PV+ neurons exceeded that of pyramidal cells, with a systematic decrease in E/I transfer impedance ratio with increasing stimulation frequency (Figures 5C and 5D).

Reducing the membrane capacitance of pyramidal cell dendrites resulted in an increase in transfer impedance (Figure S5).

To test the capacitive filtering effects of dendrites in a more realistic scenario, including the filtering properties of synapses, we examined how synaptic bursts with different inter-spike intervals arriving at a dendrite translate to voltage fluctuations in the soma (Figure 5E). Specifically, we placed one synapse at a dendrite 150 μm from the soma and varied the frequency of the synaptic input between 1 and 100 Hz. We found that the transfer impedance during synaptic burst stimulation was, in general, higher for PV+ than for pyramidal cells, especially for higher frequencies (Figure 5F). Again, we observed a systematic decrease in the transfer-impedance ratio between excitatory and inhibitory cells (Figure 5G).

Finally, we wanted to test the differences in the spike phase locking between the cell types, i.e., examine the supra-threshold firing behavior and its relation to rhythmic stimulation. We reasoned that in the experimental data, V1 neurons receive both rhythmic bottom-up inputs from the LGN as well as background synaptic inputs that are uncorrelated to the rhythmic stimulation. Accordingly, in our simulations, we used two sets of synaptic inputs at the dendrites of the simulated neurons: a first subset of synapses, placed along the whole dendritic tree, was activated using homogeneous Poisson spike trains, which mimicked the uncorrelated (recurrent) background activity of V1 cells in the flickering experiment. In addition, we placed a second set of synapses at the basal dendrites of pyramidal cells and along the entire dendritic tree of PV+ cells. The latter synapses were activated by an inhomogeneous Poisson process (modulated at frequencies between 10 and 80 Hz) to simulate rhythmic bottom-up input from the LGN to V1 during visual flicker stimulation (Figures 5H and 5I). The modulation strength of the inhomogeneous Poisson input spike trains was adjusted to match the phase locking of the recorded neurons in the LGN during a 10 Hz flicker stimulation (Figure S4A). The number of synapses and synaptic weights was fitted to reproduce the firing rates and phase locking

CellPress
OPEN ACCESS

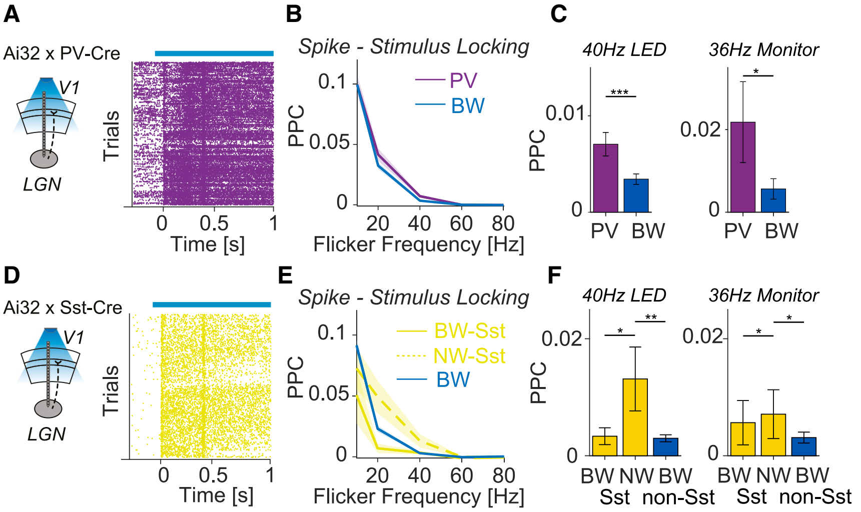

Figure 4. Spike-stimulus phase locking of GABAergic subtypes

(A) Illustration of the experimental setup in PValb-IRES-Cre mice. PV+ cells were identified by their modulation of firing during the stimulation of V1 using a blue laser (see STAR Methods).

(B) PPC between spikes of PV+ (n = 152) as well as BW (n = 525) neurons and the LED flicker stimulus.

(C) Phase locking of PV+ and BW neurons during 40 Hz LED (left, $n_{PV+} = 152$, $n_{BW} = 525$) and 36 Hz monitor (right, $n_{PV+} = 80$, $n_{BW} = 104$) stimulation. ***$p < 0.001$; **$p < 0.01$; *$p < 0.05$, non-parametric permutation tests, based on 1,000 randomizations.

(D) Illustration of the experimental setup in SOM-IRES-Cre mice. Sst+ cells were identified by their modulation in firing during the stimulation of V1 using a blue laser (see STAR Methods).

(E) PPC between spikes of NW Sst+ and BW Sst+ as well as BW neurons and the LED flicker stimulus.

(F) Phase locking of Sst+ and BW neurons during 40 Hz LED (left, $n_{BW,Sst} = 13$, $n_{NW,Sst} = 32$, $n_{BW} = 541$) and 36 Hz monitor (right, $n_{BW,Sst} = 8$, $n_{NW,Sst} = 26$, $n_{BW} = 464$) stimulation. ***$p < 0.001$; **$p < 0.01$; *$p < 0.05$, non-parametric permutation tests, based on 1,000 randomizations. Error bars and shadings indicate SEMs.

of the two neuron classes during 10 Hz LED stimuli (Figure S4B; see STAR Methods). After fitting the connectivity parameters based on the 10 Hz stimulation, we generated inhomogeneous spike trains modulated at higher frequencies (20, 40, 60, and 80 Hz). The modulation strength of the inhomogeneous input spike trains was adjusted to reproduce the observed spike sLFP phase locking in the LGN recordings (Figure S4C). Increasing the frequency of the input rhythm resulted in a substantial decrease in the phase locking of neurons (Figure 5J). This decrease in phase locking with increasing stimulation frequency was much steeper in pyramidal than in PV+ neurons, similar to the experimental findings reported above (Figure 5J). In line with the experimentally observed decrease in the E-I locking ratio with increasing stimulation frequency (Figure 3C), the relative phase locking of pyramidal cell models and PV+ models decreased with increasing stimulation frequency (Figure 5K). Increasing the dendritic membrane capacitance in pyramidal cells resulted in stronger phase locking at high frequencies, enabling pyramidal cells to follow a 40 Hz stimulation similar to PV+ cells (Figures S5C and S5D).

DISCUSSION

We investigated the propagation of rhythmic visual flicker stimulation throughout different stages of the visual hierarchy. Strong phase-locked responses in LGN neurons were induced by presenting flickering stimuli between 10 and 80 Hz using either LEDs or a monitor. LGN neurons showed strong phase locking at flicker frequencies up to 40 Hz, whereas phase locking was substantially weaker in V1 units and absent in hippocampal CA1 units. The observed absence of phase locking in CA1 matches with a recent report showing a lack of 40 Hz entrainment of CA1 neurons in mice Alzheimer models by Soula et al.[33] Separating neurons in the different layers of V1 revealed an attenuation of phase locking at each processing stage of V1, especially at gamma frequencies. Phase locking was strongest in the input layer and substantially weaker in the superficial layers. Stimuli flickering in the gamma-frequency range predominantly caused phase locking in neurons with a narrow action-potential waveform, which is characteristic of fast-spiking interneurons. Optogenetic-tagging experiments showed that PV+ and NW Sst+ neurons exhibit substantially stronger phase locking to high-frequency stimuli compared with excitatory BW neurons or BW Sst+ neurons. Finally, a computational model could explain the observed differences in phase locking based on the neurons' capacitative low-pass filtering properties.

Differences in cell types

We observed that the impact of flicker-induced synchrony differed substantially between cell types: flickering stimuli in the gamma-frequency range predominantly caused phase locking in NW fast-spiking interneurons. Optogenetic labeling experiments demonstrated that PV+ and NW Sst+ exhibited much stronger phase locking to high-frequency stimuli compared with excitatory BW or BW Sst+ neurons. This observation is consistent with the well-known low-pass filtering properties of excitatory cells.[19–22] By contrast, the fast kinetics of PV+ interneurons enable them to respond to input signals with high temporal precision.[34–39] However, our results differ from previous work also in one important respect: we did not observe suprathreshold gamma-frequency resonance in V1 interneurons, in contrast to some previous studies.[21,40]

Compared with PV+ neurons, Sst+ interneurons are associated with slower signaling due to their intrinsic biophysical properties with relatively broad spikes and slow membrane time constants,[34–36,41,42] which matches with our observation of weak phase locking in BW Sst+ neurons. Consistent with our results, previous studies have shown that Sst+ cells are a relatively heterogeneous cell class in terms of electrophysiological properties and include a subtype with BW spikes and one with NW spikes.[34,43] Previous studies have suggested that NW Sst+ neurons mainly correspond to non-Martinotti cells that have lower input resistance and higher firing rates than Martinotti cells, which tend to have BW action potentials.[44–46] This difference

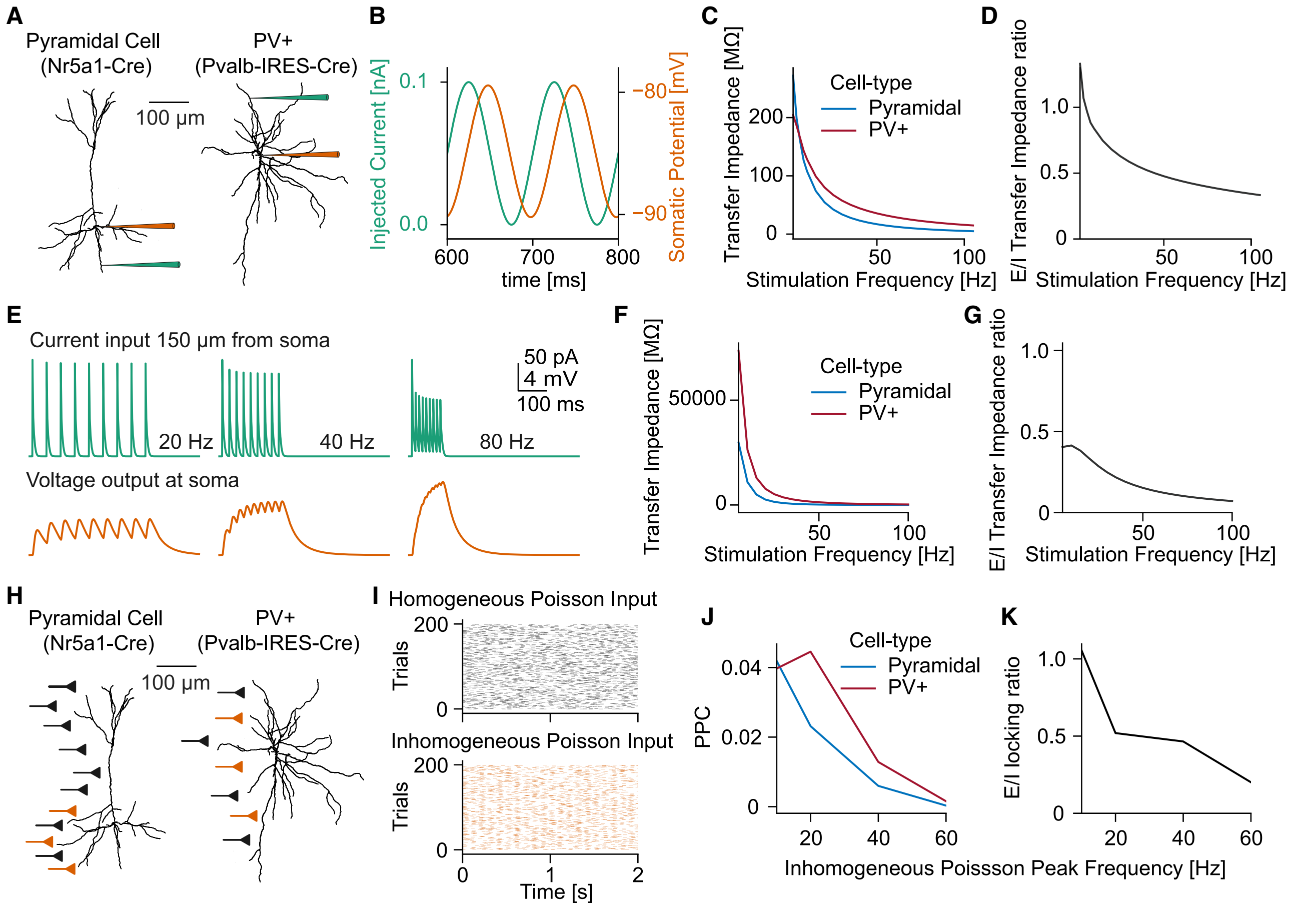

Figure 5. Filtering properties of V1 multicompartmental neuron models

(A) Illustration of current-clamp experiment on N5a1-Cre pyramidal neuron and PVAL-IRES-Cre neuron.

(B) Somatic voltage fluctuations (orange) during sinusoidal current injection into the dendrite at 150 μm (green).

(C) Transfer impedance of PV+ and pyramidal cell model during sinusoidal stimulation between 1 and 100 Hz.

(D) Ratio between the transfer impedance of pyramidal (excitatory) and PV+ (inhibitory) neurons.

(E) Synaptic input current 150 μm from the soma (green, top) and somatic voltage response (orange, bottom).

(F) Transfer impedance of PV+ and pyramidal cell model during synaptic burst stimulation between 1 and 100 Hz.

(G) Ratio between the transfer impedance of pyramidal (excitatory) and PV+ (inhibitory) neurons during synaptic burst stimulation.

(H) Illustration of synaptic stimulation with homogeneous and inhomogeneous Poisson input.

(I) Raster plot of example neurons from homogeneous (black) and inhomogeneous (orange) Poisson spiking input population.

(J) Phase locking of pyramidal (blue) and PV+ (red) to sLFP of inhomogeneous Poisson spiking input population.

(K) Ratio between the phase locking of pyramidal (excitatory) and PV+ (inhibitory) cell models.

in properties between BW and NW Sst+ neurons matches our observation that only the NW Sst+ neurons showed prominent phase locking to higher-frequency flicker stimuli.

Our findings generally agree with the idea that PV+, rather than Sst+, neurons may be involved in the generation of high gamma-frequency oscillations in the cortex.[47–51] For instance, Cardin et al.[39] used optogenetic stimulation of PV+ and pyramidal cells at various frequencies in mouse S1. In congruence with our observation, they found that stimulating PV+ cells selectively amplifies gamma oscillations, while pyramidal cells only amplify low-frequency stimulation.[39] Tiesinga[52] was able to reproduce these results in a model in which pyramidal cells are endowed with a slow hyperpolarizing current, leading to an intrinsic preference for low frequencies by pyramidal cells. Furthermore, Chen et al.[51] have shown different frequency preferences of Sst+ and PV+ cells, with Sst+ preferentially synchronizing in a narrow low-frequency range (6–30 Hz) and PV+ preferentially synchronizing in a broader high-frequency range (20–80 Hz). Interestingly, we did not find significant phase locking in BW Sst+ interneurons around 20 Hz, in contrast to NW Sst+ interneurons. It is unclear how to reconcile this observation with the hypothesis that Sst+ interneurons play an important role in the V1 PING (pyramidal interneuron gamma) circuit that generates low gamma-frequency oscillations (~30 Hz) during visual stimulation.[53] A possible explanation, which remains to be tested, is that NW Sst+ interneurons, rather than BW Sst+ interneurons, show precise phase locking during low gamma-frequency oscillations.

Propagation of flicker-induced synchrony

Our findings generally fit well with temporal low-pass filtering properties of signals between the LGN and different layers of V1, as observed using drifting grating stimulation in anesthetized macaque monkeys.[54] In various species (cats, tree shrews, monkeys, humans), studies have measured neural activity in response to visual flickering stimuli. These studies have observed a similar trend, namely a decrease in phase locking with increasing stimulation frequency, with the strongest phase locking occurring in neurons in the input layer of V1.[55–57] Our present study goes beyond this previous work in two important aspects.

(1) We used high-density probes to record from multiple processing levels (layers, areas) simultaneously (as opposed to previous studies), which allowed us to look at inter-areal correlations and progressive changes in modulation. Using this approach, we conclude that at high frequencies, there is a major decrease in phase locking from the LGN to L4 of V1, another major decrease from L4 to L2/3 of V1, and a lack of phase locking at the level of the hippocampus. Because we performed simultaneous recordings from the LGN, we could verify that phase locking of V1 neurons to the visual stimulus itself was comparable to the phase locking of V1 neurons to the population LGN signal (the sLFP). That is, quantifying phase locking of V1 neurons to the monitor did not miss endogenous synchronization that departs from the visual stimulus' frequency (e.g., random fluctuations in synchronization frequency around the monitor frequency). We further note that the study was performed during wakefulness, which is important because anesthesia can have strong effects on flicker-induced synchronization.[58]

(2) In contrast to previous work, we distinguished between excitatory and distinct kinds of inhibitory neurons. We observed substantially stronger phase locking in inhibitory compared with excitatory V1 neurons. Hence, it is possible that reports of phase locking in previous studies may have resulted mainly from phase locking in inhibitory interneurons. Interestingly, we found the strongest 40 Hz phase locking in LGN neurons, the vast majority of which should be excitatory. This means that there was a major (almost 2 orders of magnitude) difference in phase locking between the excitatory LGN neurons and the excitatory neurons in V1. An interesting question is which channel properties in excitatory LGN neurons allow them to follow higher-frequency inputs.

A difference between our and previous results in other species (cats, tree shrews, monkeys, humans) is the maximum frequency at which phase locking is observed.[55,57,59,60] For instance, recent MEG studies showed phase locking to flicker up to 80 Hz in human MEG experiments,[15] while we did not see any responses at 60 and 80 Hz. In line with this observed difference between mice and humans, the critical flicker fusion frequency is much lower in rodents than in humans.[61,62] This difference in the maximum frequency that the visual circuit can follow can be likely attributed to physiological differences in the visual cortex between rodents and primates, e.g., specific cell types in primates.[63] It should also be noted that visually evoked gamma oscillations in V1 are generally faster in primates than in mice.[64,65]

Another key difference between our study and human studies of frequency tagging is that we examined single-neuron spiking activity. Analysis of field potential (e.g., MEG, EEG) signals cannot determine whether spiking activity is phase locked to flicker activity. The reason is that field potential signals reflect synaptic inputs that derive from two potential sources: (1) afferent inputs from other areas and (2) local spiking activity.[28,66,67] Thus, MEG/EEG signals in V1 can be generated by spatiotemporally coherent LGN afferents driving excitatory post-synaptic potentials (EPSPs) in V1 input layer. The extent to which MEG/EEG signals reflect local spiking activity depends on the extent to which local neurons are phase locked to the afferent inputs. The coherence of MEG/EEG signals with the flicker stimulus may thus reflect thalamocortical inputs. Our results further suggest that the relative contribution of cortical spiking and thalamocortical afferents to MEG/EEG signals depends on the flicker frequency: at lower frequencies, V1 phase locking is relatively strong, and MEG/EEG signals should therefore reflect approximately equal contributions from afferent inputs and local spiking activity.[28] By contrast, at higher frequencies, V1 phase locking becomes very weak compared with LGN phase locking, which predicts that the coherence of EEG/MEG signals with the flicker stimulus is largely driven by thalamocortical afferents. Given these considerations, the attentional modulation of flicker-induced responses in human MEG[5] may reflect (1) a modulation of V1 spiking responses, (2) the efficacy of LGN to V1 inputs, or (3) attentional effects in LGN.[68]

The observation of weak propagation of high-frequency synchronization is in line with our recent findings based on endogenous oscillations in macaque and mice: Spyropoulos et al.[65] found that endogenous gamma-frequency oscillations in awake macaque V1 mainly recruit V4 fast-spiking inhibitory interneurons that reside in the granular input layer of V4, with no phase locking in superficial layers of V4. Similar observations were made for the propagation of LGN gamma synchronization to area V1 in mice.[65] As in the present study, optotagging experiments in mice further suggest that endogenous LGN gamma predominantly drives fast-spiking PV+ and Sst+ interneurons in the input layer of V1.[65] The observation that CA3 gamma synchronization primarily recruits CA1 interneurons[69] suggests a motif that may be prevalent across many cortical regions. The computational models presented here suggest that the preferential recruitment of fast-spiking interneurons in the granular layer by high-frequency afferents can be explained by differences in capacitive low-pass filtering properties. This conclusion is in line with previous studies that have examined single-neuron filtering properties in fast-spiking interneurons and excitatory neurons.[21,24,40] This difference in the filtering properties of fast-spiking interneurons and pyramidal cells may lead to a shift in the E-I balance toward inhibition under high-frequency flicker stimulation, which could in turn prevent propagation beyond the input layer.

The weak propagation of high-frequency synchronization and its effect on different cell types contradicts the hypothesis that gamma synchronization promotes feedforward information transmission.[10,14,70] We note that the evidence for this hypothesis is exclusively based on the analysis of LFP-LFP

Granger causality,[13,14] which can therefore not determine the effect of synaptic afferents on spiking activity in the downstream receiver.[28] However, our findings match with several observations: first, the observation that high-frequency oscillations are mainly locally coherent and recruit neurons mostly restricted to the area of origin, in contrast to more globally coherent slower rhythms,[16–18,71] and second, the observation that strong (endogenous) gamma synchronization in the LGN and V1 is mostly observed during the presentation of highly predictable, low-dimensional stimuli, which have, on average, a low salience.[28,72,73]

Use of flicker stimuli for frequency tagging and neurodegenerative diseases

Our findings have several implications for the use of frequency tagging as a method to track neural processing. While most studies on frequency tagging used low-frequency stimuli, recent studies suggest that using higher frequencies than the flicker fusion threshold may have two advantages: first, the use of high frequencies avoids interference of the consciously perceived flicker with the task.[8] Second, using high frequencies may avoid interference of the rhythmic stimuli with endogenous oscillations.[8] However, in the present study, we showed a clear disadvantage of using higher frequencies for frequency tagging: we observed little propagation of high-frequency synchronization beyond the granular input layer of V1 and a reduction in phase locking of about an order of magnitude per processing stage. Thus, using high frequencies might be primarily useful for tagging neural activity and studying excitability in early processing stages. This conclusion is in line with recent human EEG and MEG studies showing that strong responses at the tagged frequency are mostly restricted to early sensory areas.[5,7,15,57,74]

Flicker stimuli have also been applied in the context of neurodegenerative diseases. Recent studies have shown a significant decrease in Alzheimer-associated beta-amyloid plugs across many cortical regions (V1, PFC, CA1, S1) following chronic 40 Hz LED flicker stimulation.[1,3,75] These studies also showed significant LFP-LFP coherence between the visual cortex and higher cortical regions including the PFC and CA1, which was suggested as a potential mechanism underlying the effects of flicker on neurodegeneration.[3] Here, we did not observe phase locking of single CA1 neurons to the flicker stimulus, which is unlikely due to a lack of sensitivity, as we recorded from a large sample of CA1 neurons. Our findings on CA1 are consistent with a recent report that 40 Hz phase locking is absent for CA1 neurons in mice Alzheimer models.[33] Furthermore, we observed a strong attenuation of high-frequency synchronization already at early processing stages. This raises the question as to whether the previously observed V1-CA1 LFP-LFP coherence in fact reflects the phase locking of CA1 spikes. Considering that the visual cortex is located in close proximity to CA1, it is generally difficult to rule out volume conduction.[76,77] Another key difference between our study and the previous neurodegeneration study is that we did not use chronic flicker stimulation across several weeks. It is possible that long-term chronic flicker stimulation induces synaptic plasticity, which could lead to an enhancement of entrainment in higher-order brain regions across weeks. Nevertheless, it remains a possibility that the therapeutic effects of flicker stimulation on neurodegeneration in CA1 are not mediated by the phase locking of CA1 neurons. We note that a recent study failed to replicate the effects of 40 Hz flicker on neurodegeneration in mice Alzheimer models,[33] and it is at present unclear what factors determine the previously observed effects by Adaikkan et al.[3]

Limitations of the study

In this study, mice were exposed to rhythmic visual stimulation over the time course of 1 h. In contrast, studies using rhythmic flicker stimulation as a therapeutic method for neurodegenerative diseases used chronic flicker simulation over the course of days. As discussed above, it is possible that the differences in entrainment between our and previous studies at the level of CA1 can be attributed to the usage of chronic stimulation in previous studies. This would suggest that entrainment effects should increase over time, which can be tested in a straightforward manner with chronic high-density recordings. However, we note that previous work did not report phase locking of single units to the LEDs, and therefore a feasible experiment is to first perform chronic flicker followed by acute recordings. Related to this, we note that since we sampled from only a subset of CA1 cells, we cannot exclude the possibility of phase-locked neurons in other hippocampal subfields (e.g., dentate gyrus), which could also contribute to CA1 LFPs.

Another question which we did not explore was the relationship between the spatial structure of the stimulus and flicker.[15] It is possible that the exposure to structured flickering stimuli that maximally drive V1 (e.g., certain natural images) would lead to more widespread entrainment of neurons in V1. It should be noted, however, that we have made similar findings with the propagation of endogenous gamma oscillations in mice and macaques, e.g., the propagation from V1 to V4 with grating stimuli.[65]

We observed a substantial fraction of NW Sst+ neurons, consistent with previous studies, and their firing responses were in line with their biophysical properties (see above). Nevertheless, we cannot exclude that a fraction of these neurons overlap with the PV+ class.[78] Yet, this does not affect our main conclusion on the main comparison between excitatory, BW Sst+, and fast-spiking PV+ interneurons.

In this study, we did not systematically investigate the effect of luminance on phase locking. We note, however, that we used high LED luminances (as in previous work[1]) and standard monitor luminances. As the luminance of the LED exceeded the one of the monitor, it is possible that this accounts for stronger observed phase locking for the LED stimuli. It is also possible that fast-spiking interneurons and excitatory neurons may have a different dependence of phase locking on stimulus intensity. However, a systematic investigation of the dependence of phase locking on luminance needs to be undertaken in future work.

We note that the constructed sLFP signal (based on all recorded spikes) in the LGN might not be a perfect proxy of the population activity in the LGN. By generating the sLFP signal from all recorded neurons in the LGN, we have included different assemblies (e.g., ON and OFF neurons) that could blur the overall signal due to their time-shifted activity. Nevertheless, since the locking of cells to the stimulus was very similar to the locking

to the LGN sLFP signal, our main conclusions should not be affected, as they hold true for phase locking to the monitor.

Conclusions

We show here how synchronized activity, induced by flicker stimuli, propagates across brain areas and affects distinct cell types depending on the frequency. Specifically, our findings suggest that low-frequency synchronization propagates effectively across multiple cortical stages and recruits excitatory and inhibitory neurons to a similar extent, whereas high-frequency synchronization tends to stay local and primarily recruits fast-spiking interneurons downstream. This could suggest that when a neural population switches from low- to high-frequency synchronization, output signals will be differently integrated by a downstream receiver. These findings have several implications for the understanding of frequency tagging methods and the mechanisms underlying the effects of high-frequency stimulation on neurodegeneration.

STAR★METHODS

Detailed methods are provided in the online version of this paper and include the following:

- KEY RESOURCES TABLE
- RESOURCE AVAILABILITY
  - Lead contact
  - Materials availability
  - Data and code availability
- EXPERIMENTAL MODEL AND SUBJECT DETAILS
  - Flicker stimulation
- METHOD DETAILS
  - Neuropixels recordings and optogenetics
  - Multi-compartmental models
- QUANTIFICATION AND STATISTICAL ANALYSIS
  - Identification of CA1 pyramidal cell layer
  - Waveform classification
  - Assignment of cortical layers in V1
  - Testing optogenetic response
  - Testing visual responsive neurons
  - Statistical modeling
  - Statistical testing

SUPPLEMENTAL INFORMATION

Supplemental information can be found online at https://doi.org/10.1016/j.celrep.2023.112492.

ACKNOWLEDGMENTS

This project was supported by ERC Starting Grant to M.V. (SPATEMP) and a BMF Grant to M.V. (Bundesministerium fuer Bildung und Forschung, Computational Life Sciences, project BINDA, 031L0167). We thank Paul Tiesinga for helpful comments and insightful discussions.

AUTHOR CONTRIBUTIONS

Conceptualization, M.S. and M.V.; simulations and data analysis, M.S.; mouse recordings and surgeries, A.T.; software and methods for mouse data, M.S., A.T., C.U., and M.V.; writing, M.S. and M.V.; supervision, M.V.

DECLARATION OF INTERESTS

The authors declare no competing interests.

Received: January 8, 2023
Revised: March 10, 2023
Accepted: April 24, 2023
Published: May 16, 2023

REFERENCES

1. Singer, A.C., Martorell, A.J., Douglas, J.M., Abdurrob, F., Attokaren, M.K., Tipton, J., Mathys, H., Adaikkan, C., and Tsai, L.-H. (2018). Noninvasive 40-hz light flicker to recruit microglia and reduce amyloid beta load. Nat. Protoc. *13*, 1850–1868. https://doi.org/10.1038/s41596-018-0021-x.
2. Adaikkan, C., and Tsai, L.-H. (2020). Gamma entrainment: impact on neurocircuits, glia, and therapeutic opportunities. Trends Neurosci. *43*, 24–41. https://doi.org/10.1016/j.tins.2019.11.001.
3. Adaikkan, C., Middleton, S.J., Marco, A., Pao, P.-C., Mathys, H., Kim, D.N.-W., Gao, F., Young, J.Z., Suk, H.-J., Boyden, E.S., et al. (2019). Gamma entrainment binds higher-order brain regions and offers neuroprotection. Neuron *102*, 929–943.e8. https://doi.org/10.1016/j.neuron.2019.04.011.
4. Norcia, A.M., Appelbaum, L.G., Ales, J.M., Cottereau, B.R., and Rossion, B. (2015). The steady-state visual evoked potential in vision research: a review. J. Vis. *15*, 4. https://doi.org/10.1167/15.6.4.
5. Zhigalov, A., Herring, J.D., Herpers, J., Bergmann, T.O., and Jensen, O. (2019). Probing cortical excitability using rapid frequency tagging. Neuroimage *195*, 59–66. https://doi.org/10.1016/j.neuroimage.2019.03.056.
6. Vialatte, F.-B., Maurice, M., Dauwels, J., and Cichocki, A. (2010). Steady-state visually evoked potentials: focus on essential paradigms and future perspectives. Prog. Neurobiol. *90*, 418–438. https://doi.org/10.1016/j.pneurobio.2009.11.005.
7. Drijvers, L., Jensen, O., and Spaak, E. (2021). Rapid invisible frequency tagging reveals nonlinear integration of auditory and visual information. Hum. Brain Mapp. *42*, 1138–1152. https://doi.org/10.1002/hbm.25282.
8. Seijdel, N., Marshall, T.R., and Drijvers, L. (2023). Rapid invisible frequency tagging (RIFT): a promising technique to study neural and cognitive processing using naturalistic paradigms. Cereb. Cortex *33*, 1626–1629. https://doi.org/10.1093/cercor/bhac160.
9. Bernander, O., Koch, C., and Douglas, R.J. (1994). Amplification and linearization of distal synaptic input to cortical pyramidal cells. J. Neurophysiol. *72*, 2743–2753. https://doi.org/10.1152/jn.1994.72.6.2743.
10. Salinas, E., and Sejnowski, T.J. (2001). Correlated neuronal activity and the flow of neural information. Nat. Rev. Neurosci. *2*, 539–550. https://doi.org/10.1038/35086012.
11. Fries, P., Reynolds, J.H., Rorie, A.E., and Desimone, R. (2001). Modulation of oscillatory neuronal synchronization by selective visual attention. Science *291*, 1560–1563. https://doi.org/10.1126/science.1055465.
12. Bruno, R.M., and Sakmann, B. (2006). Cortex is driven by weak but synchronously active thalamocortical synapses. Science *312*, 1622–1627. https://doi.org/10.1126/science.1124593.
13. Van Kerkoerle, T., Self, M.W., Dagnino, B., Gariel-Mathis, M.-A., Poort, J., Van Der Togt, C., and Roelfsema, P.R. (2014). Alpha and gamma oscillations characterize feedback and feedforward processing in monkey visual cortex. Proc. Natl. Acad. Sci. USA *111*, 14332–14341. https://doi.org/10.1073/pnas.140277311.
14. Bastos, A.M., Vezoli, J., Bosman, C.A., Schoffelen, J.-M., Oostenveld, R., Dowdall, J.R., De Weerd, P., Kennedy, H., and Fries, P. (2015). Visual areas exert feedforward and feedback influences through distinct frequency channels. Neuron *85*, 390–401. https://doi.org/10.1016/j.neuron.2014.12.018.
15. Duecker, K., Gutteling, T.P., Herrmann, C.S., and Jensen, O. (2021). No evidence for entrainment: endogenous gamma oscillations and rhythmic

flicker responses coexist in visual cortex. J. Neurosci. *41*, 6684–6698. https://doi.org/10.1523/JNEUROSCI.3134-20.2021.

16. Steriade, M. (2001). Impact of network activities on neuronal properties in corticothalamic systems. J. Neurophysiol. *86*, 1–39. https://doi.org/10.1152/jn.2001.86.1.1.
17. Csicsvari, J., Jamieson, B., Wise, K.D., and Buzsáki, G. (2003). Mechanisms of gamma oscillations in the hippocampus of the behaving rat. Neuron *37*, 311–322. https://doi.org/10.1016/S0896-6273(02)01169-8.
18. Buzsáki, G., and Draguhn, A. (2004). Neuronal oscillations in cortical networks. Science *304*, 1926–1929. https://doi.org/10.1126/science.1099745.
19. Fortune, E.S., and Rose, G.J. (1997). Passive and active membrane properties contribute to the temporal filtering properties of midbrain neurons in vivo. J. Neurosci. *17*, 3815–3825. https://doi.org/10.1523/JNEUROSCI.17-10-03815.1997.
20. Koch, C. (2004). Biophysics of Computation: Information Processing in Single Neurons (Oxford University Press). https://doi.org/10.1093/oso/9780195104912.001.0001.
21. Pike, F.G., Goddard, R.S., Suckling, J.M., Ganter, P., Kasthuri, N., and Paulsen, O. (2000). Distinct frequency preferences of different types of rat hippocampal neurones in response to oscillatory input currents. J. Physiol. *529*, 205–213. https://doi.org/10.1111/j.1469-7793.2000.00205.x.
22. Vaidya, S.P., and Johnston, D. (2013). Temporal synchrony and gamma-to-theta power conversion in the dendrites of ca1 pyramidal neurons. Nat. Neurosci. *16*, 1812–1820. https://doi.org/10.1038/nn.3562.
23. Hutcheon, B., and Yarom, Y. (2000). Resonance, oscillation and the intrinsic frequency preferences of neurons. Trends Neurosci. *23*, 216–222. https://doi.org/10.1016/S0166-2236(00)01547-2.
24. Izhikevich, E.M., Desai, N.S., Walcott, E.C., and Hoppensteadt, F.C. (2003). Bursts as a unit of neural information: selective communication via resonance. Trends Neurosci. *26*, 161–167. https://doi.org/10.1016/S0166-2236(03)00034-1.
25. Blankenburg, S., Wu, W., Lindner, B., and Schreiber, S. (2015). Information filtering in resonant neurons. J. Comput. Neurosci. *39*, 349–370. https://doi.org/10.1007/s10827-015-0580-6.
26. Vinck, M., Battaglia, F.P., Womelsdorf, T., and Pennartz, C. (2012). Improved measures of phase-coupling between spikes and the local field potential. J. Comput. Neurosci. *33*, 53–75. https://doi.org/10.1007/s10827-011-0374-4.
27. Okun, M., Steinmetz, N., Cossell, L., Iacaruso, M.F., Ko, H., Barthó, P., Moore, T., Hofer, S.B., Mrsic-Flogel, T.D., Carandini, M., et al. (2015). Diverse coupling of neurons to populations in sensory cortex. Nature *521*, 511–515. https://doi.org/10.1038/nature14273.
28. Schneider, M., Broggini, A.C., Dann, B., Tzanou, A., Uran, C., Sheshadri, S., Scherberger, H., and Vinck, M. (2021). A mechanism for inter-areal coherence through communication based on connectivity and oscillatory power. Neuron *109*, 4050–4067.e12. https://doi.org/10.1016/j.neuron.2021.09.037.
29. Senzai, Y., Fernandez-Ruiz, A., and Buzsáki, G. (2019). Layer-specific physiological features and interlaminar interactions in the primary visual cortex of the mouse. Neuron *101*, 500–513.e5. https://doi.org/10.1016/j.neuron.2018.12.009.
30. Gouwens, N.W., Berg, J., Feng, D., Sorensen, S.A., Zeng, H., Hawrylycz, M.J., Koch, C., and Arkhipov, A. (2018). Systematic generation of biophysically detailed models for diverse cortical neuron types. Nat. Commun. *9*, 710. https://doi.org/10.1038/s41467-017-02718-3.
31. Arkhipov, A., Gouwens, N.W., Billeh, Y.N., Gratiy, S., Iyer, R., Wei, Z., Xu, Z., Abbasi-Asl, R., Berg, J., Buice, M., et al. (2018). Visual physiology of the layer 4 cortical circuit in silico. PLoS Comput. Biol. *14*, e1006535. https://doi.org/10.1371/journal.pcbi.1006535.
32. Billeh, Y.N., Cai, B., Gratiy, S.L., Dai, K., Iyer, R., Gouwens, N.W., Abbasi-Asl, R., Jia, X., Siegle, J.H., Olsen, S.R., et al. (2020). Systematic integration of structural and functional data into multi-scale models of mouse primary visual cortex. Neuron *106*, 388–403.e18. https://doi.org/10.1016/j.neuron.2020.01.040.
33. Soula, M., Martín-Ávila, A., Zhang, Y., Dhingra, A., Nitzan, N., Sadowski, M.J., Gan, W.-B., and Buzsáki, G. (2023). Forty-hertz light stimulation does not entrain native gamma oscillations in alzheimer's disease model mice. Nat. Neurosci. *26*, 1–9. https://doi.org/10.1038/s41593-023-01270-2.
34. Kvitsiani, D., Ranade, S., Hangya, B., Taniguchi, H., Huang, Z.J., and Kepecs, A. (2013). Distinct behavioural and network correlates of two interneuron types in prefrontal cortex. Nature *498*, 363–366. https://doi.org/10.1038/nature12176.
35. Lazarus, M.S., and Huang, Z.J. (2011). Distinct maturation profiles of perisomatic and dendritic targeting gabaergic interneurons in the mouse primary visual cortex during the critical period of ocular dominance plasticity. J. Neurophysiol. *106*, 775–787. https://doi.org/10.1152/jn.00729.2010.
36. Beierlein, M., Gibson, J.R., and Connors, B.W. (2003). Two dynamically distinct inhibitory networks in layer 4 of the neocortex. J. Neurophysiol. *90*, 2987–3000. https://doi.org/10.1152/jn.00283.2003.
37. McCormick, D.A., Connors, B.W., Lighthall, J.W., and Prince, D.A. (1985). Comparative electrophysiology of pyramidal and sparsely spiny stellate neurons of the neocortex. J. Neurophysiol. *54*, 782–806. https://doi.org/10.1152/jn.1985.54.4.782.
38. Jouhanneau, J.-S., Kremkow, J., and Poulet, J.F.A. (2018). Single synaptic inputs drive high-precision action potentials in parvalbumin expressing gaba-ergic cortical neurons in vivo. Nat. Commun. *9*, 1540. https://doi.org/10.1038/s41467-018-03995-2.
39. Cardin, J.A., Carlén, M., Meletis, K., Knoblich, U., Zhang, F., Deisseroth, K., Tsai, L.-H., and Moore, C.I. (2009). Driving fast-spiking cells induces gamma rhythm and controls sensory responses. Nature *459*, 663–667. https://doi.org/10.1038/nature08002.
40. Hasenstaub, A., Shu, Y., Haider, B., Kraushaar, U., Duque, A., and McCormick, D.A. (2005). Inhibitory postsynaptic potentials carry synchronized frequency information in active cortical networks. Neuron *47*, 423–435. https://doi.org/10.1016/j.neuron.2005.06.016.
41. Hu, H., Ma, Y., and Agmon, A. (2011). Submillisecond firing synchrony between different subtypes of cortical interneurons connected chemically but not electrically. J. Neurosci. *31*, 3351–3361. https://doi.org/10.1523/JNEUROSCI.4881-10.2011.
42. Miao, Q., Yao, L., Rasch, M.J., Ye, Q., Li, X., and Zhang, X. (2016). Selective maturation of temporal dynamics of intracortical excitatory transmission at the critical period onset. Cell Rep. *16*, 1677–1689. https://doi.org/10.1016/j.celrep.2016.07.013.
43. Ma, Y., Hu, H., Berrebi, A.S., Mathers, P.H., and Agmon, A. (2006). Distinct subtypes of somatostatin-containing neocortical interneurons revealed in transgenic mice. J. Neurosci. *26*, 5069–5082. https://doi.org/10.1523/JNEUROSCI.0661-06.2006.
44. Scala, F., Kobak, D., Shan, S., Bernaerts, Y., Laturnus, S., Cadwell, C.R., Hartmanis, L., Froudarakis, E., Castro, J.R., Tan, Z.H., et al. (2019). Layer 4 of mouse neocortex differs in cell types and circuit organization between sensory areas. Nat. Commun. *10*, 4174. https://doi.org/10.1038/s41467-019-12058-z.
45. Nigro, M.J., Hashikawa-Yamasaki, Y., and Rudy, B. (2018). Diversity and connectivity of layer 5 somatostatin-expressing interneurons in the mouse barrel cortex. J. Neurosci. *38*, 1622–1633. https://doi.org/10.1523/JNEUROSCI.2415-17.2017.
46. Muñoz, W., Tremblay, R., and Rudy, B. (2014). Channelrhodopsin-assisted patching: in vivo recording of genetically and morphologically identified neurons throughout the brain. Cell Rep. *9*, 2304–2316. https://doi.org/10.1016/j.celrep.2014.11.042.
47. Vinck, M., Womelsdorf, T., and Fries, P. (2013). In Principles of Neural Coding, R. Quiroga-Quian and S. Panzeri, eds. (CRC Press).
48. Vinck, M., Womelsdorf, T., Buffalo, E.A., Desimone, R., and Fries, P. (2013). Attentional modulation of cell-class-specific gamma-band

synchronization in awake monkey area v4. Neuron *80*, 1077–1089. https://doi.org/10.1016/j.neuron.2013.08.019.

49. Buzsáki, G., Anastassiou, C.A., and Koch, C. (2012). The origin of extracellular fields and currents–EEG, ECoG, LFP and spikes. Nat. Rev. Neurosci. *13*, 407–420.
50. Wang, X.-J. (2010). Neurophysiological and computational principles of cortical rhythms in cognition. Physiol. Rev. *90*, 1195–1268. https://doi.org/10.1152/physrev.00035.2008.
51. Chen, G., Zhang, Y., Li, X., Zhao, X., Ye, Q., Lin, Y., Tao, H.W., Rasch, M.J., and Zhang, X. (2017). Distinct inhibitory circuits orchestrate cortical beta and gamma band oscillations. Neuron *96*, 1403–1418.e6. https://doi.org/10.1016/j.neuron.2017.11.033.
52. Tiesinga, P.H.E. (2012). Motifs in health and disease: the promise of circuit interrogation by optogenetics. Eur. J. Neurosci. *36*, 2260–2272. https://doi.org/10.1111/j.1460-9568.2012.08186.x.
53. Veit, J., Hakim, R., Jadi, M.P., Sejnowski, T.J., and Adesnik, H. (2017). Cortical gamma band synchronization through somatostatin interneurons. Nat. Neurosci. *20*, 951–959. https://doi.org/10.1038/nn.4562.
54. Hawken, M.J., Shapley, R.M., and Grosof, D.H. (1996). Temporal-frequency selectivity in monkey visual cortex. Vis. Neurosci. *13*, 477–492. https://doi.org/10.1017/s0952523800008154.
55. Veit, J., Bhattacharyya, A., Kretz, R., and Rainer, G. (2011). Neural response dynamics of spiking and local field potential activity depend on crt monitor refresh rate in the tree shrew primary visual cortex. J. Neurophysiol. *106*, 2303–2313. https://doi.org/10.1152/jn.00388.2011.
56. Rager, G., and Singer, W. (1998). The response of cat visual cortex to flicker stimuli of variable frequency. Eur. J. Neurosci. *10*, 1856–1877. https://doi.org/10.1046/j.1460-9568.1998.00197.x.
57. Williams, P.E., Mechler, F., Gordon, J., Shapley, R., and Hawken, M.J. (2004). Entrainment to video displays in primary visual cortex of macaque and humans. J. Neurosci. *24*, 8278–8288. https://doi.org/10.1523/JNEUROSCI.2716-04.2004.
58. Neuenschwander, S., Rosso, G., Branco, N., Freitag, F., Tehovnik, E.J., Schmidt, K.E., and Baron, J. (2022). On the functional role of gamma synchronization in the retinogeniculate system of the cat. Preprint at bioRxiv. https://doi.org/10.1101/2022.07.25.501474.
59. Krolak-Salmon, P., Hénaff, M.A., Tallon-Baudry, C., Yvert, B., Guénot, M., Vighetto, A., Mauguière, F., and Bertrand, O. (2003). Human lateral geniculate nucleus and visual cortex respond to screen flicker. Ann. Neurol. *53*, 73–80. https://doi.org/10.1002/ana.10403.
60. Wollman, D.E., and Palmer, L.A. (1995). Phase locking of neuronal responses to the vertical refresh of computer display monitors in cat lateral geniculate nucleus and striate cortex. J. Neurosci. Methods *60*, 107–113. https://doi.org/10.1016/0165-0270(94)00226-7.
61. Goldzband, M.G., and Clark, G. (1955). Flicker fusion in the rat. J. Genet. Psychol. *87*, 257–264. https://doi.org/10.1080/00221325.1955.10532938.
62. Mankowska, N.D., Marcinkowska, A.B., Waskow, M., Sharma, R.I., Kot, J., and Winklewski, P.J. (2021). Critical flicker fusion frequency: a narrative review. Medicina *57*, 1096. https://doi.org/10.3390/medicina57101096.
63. Onorato, I., Neuenschwander, S., Hoy, J., Lima, B., Rocha, K.-S., Broggini, A.C., Uran, C., Spyropoulos, G., Klon-Lipok, J., Womelsdorf, T., et al. (2020). A distinct class of bursting neurons with strong gamma synchronization and stimulus selectivity in monkey v1. Neuron *105*, 180–197.e5. https://doi.org/10.1016/j.neuron.2019.09.039.
64. Stauch, B.J., Peter, A., Ehrlich, I., Nolte, Z., and Fries, P. (2022). Human visual gamma for color stimuli. Elife *11*, e75897. https://doi.org/10.7554/eLife.75897.
65. Spyropoulos, G., Schneider, M., van Kempen, J., Gieselmann, M.A., Thiele, A., and Vinck, M. (2023). Distinct feedforward and feedback pathways for cell-type specific attention effects. Preprint at bioRxiv. https://doi.org/10.1101/2022.11.04.515185.
66. Buzsáki, G., and Schomburg, E.W. (2015). What does gamma coherence tell us about inter-regional neural communication? Nat. Neurosci. *18*, 484–489. https://doi.org/10.1038/nn.3952.
67. Pesaran, B., Vinck, M., Einevoll, G.T., Sirota, A., Fries, P., Siegel, M., Truccolo, W., Schroeder, C.E., and Srinivasan, R. (2018). Investigating large-scale brain dynamics using field potential recordings: analysis and interpretation. Nat. Neurosci. *21*, 903–919. https://doi.org/10.1038/s41593-018-0171-8.
68. O'Connor, D.H., Fukui, M.M., Pinsk, M.A., and Kastner, S. (2002). Attention modulates responses in the human lateral geniculate nucleus. Nat. Neurosci. *5*, 1203–1209. https://doi.org/10.1038/nn957.
69. Schomburg, E.W., Fernández-Ruiz, A., Mizuseki, K., Berényi, A., Anastassiou, C.A., Koch, C., and Buzsáki, G. (2014). Theta phase segregation of input-specific gamma patterns in entorhinal-hippocampal networks. Neuron *84*, 470–485. https://doi.org/10.1016/j.neuron.2014.08.051.
70. Fries, P. (2015). Rhythms for cognition: communication through coherence. Neuron *88*, 220–235. https://doi.org/10.1016/j.neuron.2015.09.034.
71. Buzsáki, G., and Silva, F.L.d. (2012). High frequency oscillations in the intact brain. Prog. Neurobiol. *98*, 241–249. https://doi.org/10.1016/j.pneurobio.2012.02.004.
72. Uran, C., Peter, A., Lazar, A., Barnes, W., Klon-Lipok, J., Shapcott, K.A., Roese, R., Fries, P., Singer, W., and Vinck, M. (2022). Predictive coding of natural images by v1 firing rates and rhythmic synchronization. Neuron *110*, 1240–1257.e8. https://doi.org/10.1016/j.neuron.2022.01.002.
73. Vinck, M., Uran, C., and Canales-Johnson, A. (2022). The neural dynamics of feedforward and feedback interactions in predictive processing. Preprint at arXiv. https://doi.org/10.31234/osf.io/n3afb.
74. Marshall, T.R., Ruesseler, M., Hunt, L.T., and O'Reilly, J.X. (2022). The representation of priors and decisions in parietal cortex. Preprint at bioRxiv. https://doi.org/10.1101/2021.05.03.442155.
75. Iaccarino, H.F., Singer, A.C., Martorell, A.J., Rudenko, A., Gao, F., Gillingham, T.Z., Mathys, H., Seo, J., Kritskiy, O., Abdurrob, F., et al. (2016). Gamma frequency entrainment attenuates amyloid load and modifies microglia. Nature *540*, 230–235. https://doi.org/10.1038/nature20587.
76. Sirota, A., Montgomery, S., Fujisawa, S., Isomura, Y., Zugaro, M., and Buzsáki, G. (2008). Entrainment of neocortical neurons and gamma oscillations by the hippocampal theta rhythm. Neuron *60*, 683–697. https://doi.org/10.1016/j.neuron.2008.09.014.
77. Vinck, M., and Bosman, C.A. (2016). More gamma more predictions: gamma-synchronization as a key mechanism for efficient integration of classical receptive field inputs with surround predictions. Front. Syst. Neurosci. *10*, 35. https://doi.org/10.3389/fnsys.2016.00035.
78. Hu, H., Cavendish, J.Z., and Agmon, A. (2013). Not all that glitters is gold: off-target recombination in the somatostatin–ires-cre mouse line labels a subset of fast-spiking interneurons. Front. Neural Circuits *7*, 195. https://doi.org/10.3389/fncir.2013.00195.
79. Oostenveld, R., Fries, P., Maris, E., and Schoffelen, J.-M. (2011). Fieldtrip: open source software for advanced analysis of meg, eeg, and invasive electrophysiological data. Comput. Intell. Neurosci., 156869. https://doi.org/10.1155/2011/156869.
80. Steinmetz, N.A., Aydin, C., Lebedeva, A., Okun, M., Pachitariu, M., Bauza, M., Beau, M., Bhagat, J., Böhm, C., Broux, M., et al. (2021). Neuropixels 2.0: a miniaturized high-density probe for stable, long-term brain recordings. Science *372*, eabf4588. https://doi.org/10.1126/science.abf4588.
81. Hines, M.L., and Carnevale, N.T. (1997). The neuron simulation environment. Neural Comput. *9*, 1179–1209. https://doi.org/10.1162/neco.1997.9.6.1179.
82. Dai, K., Gratiy, S.L., Billeh, Y.N., Xu, R., Cai, B., Cain, N., Rimehaug, A.E., Stasik, A.J., Einevoll, G.T., Mihalas, S., et al. (2020). Brain modeling toolkit: an open source software suite for multiscale modeling of brain circuits. PLoS Comput. Biol. *16*, e1008386. https://doi.org/10.1371/journal.pcbi.1008386.

83. Brainard, D.H., and Vision, S. (1997). The psychophysics toolbox. Spat. Vis. *10*, 433–436.

84. Nandi, A., Chartrand, T., Van Geit, W., Buchin, A., Yao, Z., Lee, S.Y., Wei, Y., Kalmbach, B., Lee, B., Lein, E., et al. (2022). Single-neuron models linking electrophysiology, morphology, and transcriptomics across cortical cell types. Cell Rep. *40*, 111176. https://doi.org/10.1016/j.celrep.2022.111176.

85. Mizuseki, K., Diba, K., Pastalkova, E., and Buzsáki, G. (2011). Hippocampal ca1 pyramidal cells form functionally distinct sublayers. Nat. Neurosci. *14*, 1174–1181. https://doi.org/10.1038/nn.2894.

86. Fernández-Ruiz, A., Oliva, A., Fermino de Oliveira, E., Rocha-Almeida, F., Tingley, D., and Buzsáki, G. (2019). Long-duration hippocampal sharp wave ripples improve memory. Science *364*, 1082–1086. https://doi.org/10.1126/science.aax0758.

87. Mitzdorf, U. (1985). Current source-density method and application in cat cerebral cortex: investigation of evoked potentials and eeg phenomena. Physiol. Rev. *65*, 37–100. https://doi.org/10.1152/physrev.1985.65.1.37.

88. Montijn, J.S., Seignette, K., Howlett, M.H., Cazemier, J.L., Kamermans, M., Levelt, C.N., and Heimel, J.A. (2021). A parameter-free statistical test for neuronal responsiveness. Elife *10*, e71969. https://doi.org/10.7554/eLife.71969.

STAR★METHODS

KEY RESOURCES TABLE

REAGENT or RESOURCE	SOURCE	IDENTIFIER
Experimental models: Organisms/strains		
$Sst^{tm2.1(cre)Zjh}$/J Mice	The Jackson Labaratory	Cat#013044; RRID: MGI:4838419
B6.Cg-Gt(ROSA)26$Sor^{tm32(CAG-COP4*H134R/EYFP)Hze}$/J Mice	The Jackson Labaratory	Cat#024109; RRID: MGI:5605716
B6.129P2-$Pvalb^{tm1(cre)Arbr}$/J Mice	The Jackson Labaratory	Cat#017320; RRID: MGI:5504648
C57BL/6JRj Mice	JANVIER LABS	Cat#SC-C57J-M; RRID: MGI:2670020
Software and algorithms		
MATLAB (version 2020a)	Mathworks	https://www.mathworks.com/
FieldTrip	Oostenveld et al.[79]	https://www.fieldtriptoolbox.org
Python version 3.6	Python Software Foundation	https://www.python.org/
Kilosort 2.5	Steinmetz et al.[80]	https://zenodo.org/record/4482749#.YSjX4I4zaUk
Neuron	Hines and Carnevale[81]	https://www.neuron.yale.edu/neuron/
Brain Modeling ToolKit (BMTK)	Dai et al.[82]	https://alleninstitute.github.io/bmtk/
ripple event detection (bz_FindRipples)	Buzsaki Lab	https://github.com/buzsakilab/buzcode
Biophysical model	This paper	https://github.com/SchneiderMarius/FlickerModel https://doi.org/10.5281/zenodo.7781198

RESOURCE AVAILABILITY

Lead contact
Further information and requests for resources should be directed to and will be fulfilled by the lead contact, Martin Vinck (martin.vinck@esi-frankfurt.de).

Materials availability
This study did not generate new unique reagents.

Data and code availability
- · Data reported in this paper will be shared by the lead contact upon request.
- · All original code has been deposited at Zenodo and is publicly available. DOIs are listed in the key resources table.
- · Any additional information is available from the lead contact upon request.

EXPERIMENTAL MODEL AND SUBJECT DETAILS

Experiments were performed on three to eight months old male mice. All procedures complied with the European Communities Council Directive 2010/63/EC and the German Law for Protection of Animals and were approved by local authorities, following appropriate ethics review. Mice were maintained on a 12/12 h light/dark cycle and recordings were performed during their dark (awake) cycle. To identify the PV-positive (PV+) and SST-positive neurons (Sst+) during electrophysiological recordings, we crossed PV-Cre-mice (B6.129P2-Pvalbtm1(cre)Arbr/J, JAX Stock 017320, The Jackson Labaratory) to Ai32(RCL-ChR2(H134R)/EYFP), JAX Stock 024109, The Jackson Labaratory) mice, and Sst-IRES-Cre mice (Ssttm2.1(cre)Zjh, JAX Stock 013044, The Jackson Labaratory) to Ai32(RCL-ChR2(H134R)/EYFP) mice, to allow Cre-dependent expression of ChR2 in PV+ (PV-ChR2) and Sst+ neurons (SST-ChR2), respectively.

Flicker stimulation
In the first set of experiments, visual flicker stimuli were generated using Psychophysics Toolbox.[83] The experiment was run on a Windows 10 and stimuli were presented on an Asus PG279Q monitor set at 144 Hz refresh rate, Racing Mode, Contrast 50%, and Brightness 25%. With these settings maximum screen luminance was measured as 146.81 cd/m^2. Square wave flicker stimulation was presented on the full screen at 4 different frequencies (16 Hz, 29 Hz, 36 Hz, and 49 Hz). In the second set of experiments,

visual flicker stimuli were presented using light-emitting diodes (LEDs) with 5 different pulse frequencies (10 Hz, 20 Hz, 40 Hz, 60 Hz, and 80 Hz). Visual flicker stimulation was generated as described by Singer et al.[1] with matching LEDs and other components. The array of LEDs was placed in front of the head-fixed mice at a distance of 17 cm emitting square wave pulses. LEDs had a correlated color temperature (CCT) of 4,000 K and an intensity of 200 lux at the head-post position measured using a Flame UV-VIS Miniature Spectrometer. The trial length for each frequency was 2s with randomized inter-stimulus intervals of 4–10 s.

METHOD DETAILS

Neuropixels recordings and optogenetics

Thirty minutes prior to the head-post surgery antibiotic (Enrofloxacin, 10 mg/kg, sc, Bayer, Leverkusen, Germany) and analgesic (Metamizole, 200 mg/kg, sc) were administered. For the anesthesia, induction mice were placed in an induction chamber and briefly exposed to isoflurane (3 % in oxygen, CP-Pharma, Burgdorf, Germany). Shortly after the anesthesia induction, the mice were fixated in a stereotaxic frame (David Kopf Instruments, Tujunga, California, USA) and the anesthesia was adjusted to 0.8–1.5 % in oxygen. To prevent corneal damage the eyes were covered with eye ointment (Bepanthen, Bayer, Leverkusen, Germany) during the procedure. A custom-made titanium head fixation bar was secured with dental cement (Super-Bond C & B, Sun Medical, Shiga, Japan) exactly above the bregma suture, while the area of the recording craniotomy (V1, AP: 1.2 mm anterior to the anterior border of the transverse sinus, ML: 2.1 to 2.5 mm) was covered with cyanoacrylate glue (Insta-Cure, Bob Smith Industries Inc, Atascadero, CA USA). Four to six days after the surgery, the animals were habituated for at least five days in the experimental conditions. The day before or the same day of the first recording session a 0.8 mm^2 craniotomy was performed above V1 (AP: 1.2 mm anterior to the anterior border of the transverse sinus, ML: 2.1 to 2.5 mm) under isoflurane anesthesia. The craniotomy was covered with silicon (Kwik-Cast, World Precision Instruments, Sarasota, USA), and the mouse was allowed to recover for at least 2 h. Recording sessions were carried out daily for a maximum of 5 days, depending on the quality of the electrophysiological signal. Awake mice were head-fixed and placed on the radial wheel apparatus. We recorded simultaneously from 384 recording sites on a single Neuropixels probe, from LGN, CA1 and V1. The probe was coated with the fluorescent dye DiI (D7757, Thermo Fisher Scientific) and was inserted in the brain tissue through the V1 craniotomy under a 15° angle. We targeted PV+ and Sst+ interneurons using PV-ChR2 and SST-ChR2 mice and activated them using optogenetic stimulation. During the optogenetic experiment, an optic fiber (Thorlabs, 200um, 0.39 NA) coupled to a diode laser (LuxX CW, 473 nm, 100 mW, Omicron-Laserage Laserprodukte GmbH, Germany) was used to illuminate V1 craniotomy. The optic fiber was positioned 0.2 mm from the probe position, just above the surface of the brain. Continuous light square pulses were applied for 500 ms interleaved by 3–6 s intervals. The light intensity on the tip of the fiber was 0.02–50 mW/mm^2.

Single units were isolated using the semi-automated spike sorting algorithm Kilosort 2.5.[80] To obtain LFPs, electrode signals were first low-pass filtered at 400 Hz and then high-pass filtered at 0.1 Hz, using a third-order Butterworth filter. In order to filter out line noise, an additional band-stop filter between 49.5 and 50.5 Hz and 99 and 101 Hz was applied. Subsequently, signals were downsampled to 1200 Hz by averaging consecutive frames. For memory reasons, only every second electrode was used for the analysis of LFP signals. The pairwise phase consistency (PPC) between spikes and stimuli and spikes and LFPs was calculated using windows of 250 ms around each spike,[26] using the ft_spiketriggeredspectrum functions in the FieldTrip SPIKE toolbox.[79] Only neurons firing at least 150 spikes were considered for the calculation of spike-LFP and spike-stimuli PPC. Because LGN is a nucleus and the neurons are not aligned, the LFP signal in LGN does not reflect the oscillatory activity of the neurons in LGN. For this reason, we used a surrogate LFP (sLFP) derived from the spiking activity of the neurons in LGN.[27,28] The sLFP was derived by summing the spikes of all individual isolated units in the LGN. Subsequently, the population spike activity was filtered between 1 and 100 Hz.

Multi-compartmental models

Simulations were carried out using NEURON[81](http://www.neuron.yale.edu) and the Brain Modeling ToolKit (BMTK).[82] The perisomatic models used in this study consist of realistic reconstructions of the dendritic trees and a wide variety of active and passive membrane mechanisms, including 10 types of ion channels placed in the soma.[30] The details of the model and how ion channel parameters were tuned based on electrophysiological recordings can be found on the website of the Allen Institute http://help.brain-map.org/display/celltypes/Documentation. We used two different models of V1 Neurons, Nr5a1 (CellID = 472451419), representing a subclass of L4 and L5 excitatory pyramidal cells in mouse V1 and PV-IRES-Cre (CellID = 471085845), representing a fast-spiking inhibitory cell class in mouse V1.[30,84]

For Figures 5A–5D the simulations ran 1400 ms with time steps of 0.001 ms. Following a preprun of 200 ms, a 1 s long 100 pA sinusoidal current (frequency range: 2–10 Hz, increment 2 Hz and 10–105 Hz, increment 5 Hz) was injected in a randomly selected dendrite (in the case of the pyramidal cell model we selected a basal dendrite) at a distance of 150 μm from the soma. Electrical transfer impedance $|Z(f)|$ was measured as the ratio of the Fourier transform of the membrane voltage in the soma to that of the current input at the dendrite.[22]

$$|z(f)| = \sqrt{(\mathrm{Re}(Z(f)))^2 + (Im(Z(f)))^2}, \qquad \text{(Equation 1)}$$

where Re(Z(f)) and Im(Z(f)) are the real and imaginary parts of the ratio of the Fourier transforms at frequency f.

For Figures 5E–5G an excitatory synapse was placed at a randomly selected dendrite (in the case of the pyramidal cell model we selected a basal dendrite) at a distance of 150 μm from the soma. Synaptic stimulation was modeled with an Exp2Syn point process with the following parameters: Synaptic rise time $\tau_1 = 1$ ms, synaptic decay time $\tau_2 = 3$ ms, reversal potential E = 0 mV, synaptic delay $\Delta T = 1$ ms, and synaptic weight $g_{syn} = 0.004$ μS. Synaptic bursts consisted out of 9 spikes spiking with a rate between 5 and 100 Hz (increment 5 Hz).

For Figures 5H–5K we placed two groups of synapses along the dendrites to reproduce the activity recorded during 10 Hz LED flicker stimulation. The first set of synapses was driven by a population of homogeneous Poisson spiking neurons and placed along the whole dendritic tree (basal and apical), simulating the continuous background input through recurrent connections. The second set of synapses, driven via a population of inhomogeneous Poisson spiking neurons (modulated at frequencies 10 Hz, 20 Hz, 40 Hz, and 60 Hz), was placed along the dendrites (for the Nr5a1-Cre model only on the basal dendrites), simulating the rhythmic drive from LGN during visual flicker stimulation. We fitted the number of inhomogeneous and homogeneous spiking neurons terminating on the two cell classes and the synaptic weights in order to reproduce the firing rates and the phase-locking to the inhomogeneous spiking input population of the broad and narrow waveform neurons during 10 Hz LED flicker stimulation. Nr5a1-Cre neurons received inhomogeneous spiking input from 6 neurons, while PV-IRES-Cre received input from 8 inhomogeneous spiking neurons. Nr5a1-Cre neurons received homogeneous spiking input from 60 neurons, while PV-IRES-Cre received input from 43 inhomogeneous spiking neurons. All input neurons had 70 synaptic connections to our model neurons with a synaptic weight of $g_{syn} = 0.000012$ μS and synaptic delay of $\Delta T = 4$ ms. Synaptic time constants were the same as in Figures 5E–5G. The simulations were run for 10 s with 200 repetitions.

QUANTIFICATION AND STATISTICAL ANALYSIS

Identification of CA1 pyramidal cell layer

The hippocampal CA1 pyramidal cell layer was identified based on several physiological criteria such as sharp wave ripples, increased single-unit activity, and large waveform amplitudes.[85,86] LFP signals from each electrode were band-pass filtered between 130 and 200 Hz followerd by a transformation to a normalized squared signal (NSS). Ripple events were identified as peaks beyond 5 SD above the mean of the normalized squared signal, with a duration between 20 ms and 200 ms. The CA1 pyramidal layer was identified as the recording site with the largest mean power during ripple events. The site with the largest spike waveform amplitude and increased spiking activity in proximity to the recording site with the largest mean ripple power was regarded as the site of CA1 pyramidal cell bodies. All physiological localizations were followed by histological verification.

The scripts for ripple event detection (bz_FindRipples) can be found at the Buzsaki lab GitHub repository https://github.com/buzsakilab/buzcode.

Waveform classification

The mean waveform was calculated over data segments from −41 to 42 samples around the time of the spike, based on the aligned waveforms of the first 10000 spikes of each neuron. The sampling rate was increased by a factor of 3 using spline interpolation. The mean waveforms were normalized by subtracting the median of the first 10 samples and then dividing by the absolute value of the negative peak. Waveforms with a positive absolute peak were discarded. Subsequently, two-dimensional t-Stochastic Neighbor Embedding (t-SNE; perplexity of 80) was applied on the 80 samples after the negative spike peak of the waveforms. Lastly, we applied hierarchical clustering on the two-dimensional t-SNE embedding, which resulted in two separate clusters corresponding to the broad and narrow waveform neurons.

Assignment of cortical layers in V1

The assignment of superficial, granular, and deep cortical layers in mouse V1 was based on the current source density (CSD) of the average LFP signal during whole screen flash stimulation. The protocol consisted of a 100 ms long white screen period with a 2 s lasting grey-screen inter-stimulus period. To increase the spatial sampling rate, LFP traces were interpolated with an interpolation factor of 4. Current source density analysis was computed by taking the second discrete spatial derivative across the different electrode recordings sites.[87] The stepsize of the discrete spatial derivative was 200 μm. Single units were assigned to a cortical layer based on the location of the channel with the highest amplitude during a spike.

Testing optogenetic response

Optogenetic tagging experiments were performed on Pvalb-IRES-Cre and Sst-IRES-Cre knock-in mice. The optogenetic stimulation consisted of 300 trials of 500 ms long stimulation periods with a randomized interstimulus interval between 3 and 6 s. Cells expressing Cre were identified using the Zeta-test.[88] The Zeta test is a recently developed parameter-free statistical test that can be used to determine whether neurons show a time-dependent modulation of their firing rates by an event. The Zeta test was applied to the period around the laser-onset (−10 ms, 10 ms) to test which neurons showed significantly modulated spiking activity ($p < 0.05$). Cells were classified as optogenetically tagged if there they exhibited a significant modulation and if their first crossing of peak half-height, occurred within the 10 ms following the onset of the laser. To avoid misclassification due to laser artifacts, neurons with a peak occurring earlier than 1 ms after the onset of the optogenetic stimulation were discarded.

Testing visual responsive neurons

The visually responsive neurons were identified using the Zeta-test on the protocol for mapping cortical layers in V1. The Zeta test was applied to the period around the onset of the white screen (0 ms,10 ms) to test which neurons showed significantly modulated spiking activity ($p < 0.05$). Cells were classified as visually responsive if their firing rate was significantly modulated by the onset of the white screen.

Statistical modeling

We fitted a regression model to predict neural spiking activity $r_{rec}(t)$ from the phase $\varphi(t)$ of the flickering stimulus using maximum likelihood estimation. The phase of the flickering stimulus was extracted by calculating the wavelet transform of the signal of a photodiode placed in front of the LEDs. The model is given by:

$$y(t) = \beta_0 \, sin(\varphi(t) + \beta_1) + \beta_2 \qquad \text{(Equation 2)}$$

where $\varphi(t)$ is the phase of the stimulus, and β_0, β_1, and β_2 are regression parameters. To estimate the firing rate $r(t)$, the model function $y(t)$ was passed through an exponential link function.

$$r(t) = e^{y(t)} \qquad \text{(Equation 3)}$$

Regression parameters were optimized by minimizing the negative log likelihood function. The performance of the model was evaluated by computing the Pearson correlation coefficient between the recorded and the predicted spike trains. Spike trains and stimulus traces were downsampled to a sampling rate of 125 Hz. Regression models were fitted using 90% of the trials of each flicker stimulation frequency and validated based on the 10% held-out trials.

Statistical testing

Statistical details, including the specific statistical tests and p values are specified in the corresponding figure legends or results section. In general, Wilcoxon Mann-Whitney test and two sided non-parametric permutation test were performed. Throughout the whole paper data are presented as the mean – SEM, unless otherwise indicated. All statistical analyses were conducted using MATLAB 2020a (Mathworks).

Cell Reports, Volume *42*

Supplemental information

Cell-type-specific propagation of visual flicker

Marius Schneider, Athanasia Tzanou, Cem Uran, and Martin Vinck

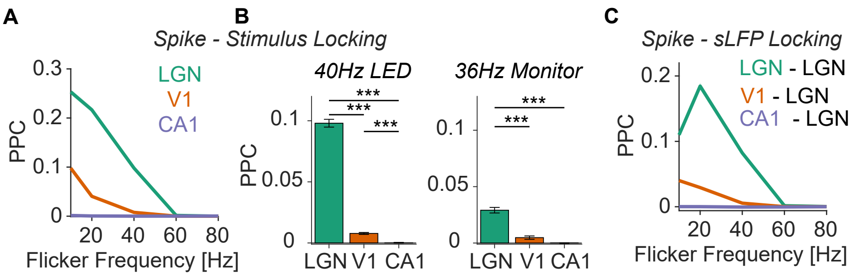

Figure S1: Same as Figure 2 but including only visually responsive neurons. (A) Spike-stimulus phase locking of visually responsive neurons in LGN (n=1332), V1 (n=1028), and CA1 (n=72) during LED flicker stimulation. **(B)** Phase locking to stimulus during 40 Hz LED (left, n_{LGN} = 1332, n_{V1} = 1028, n_{CA1} = 72) and 36 Hz monitor (right, n_{LGN} = 461, n_{V1} = 336, n_{CA1} = 16) flicker presentation. ***P<0.001; **P<0.01; *P<0.05, non-parametric permutation tests, based on 1000 randomizations. **(C)** Spike-sLFP phase locking for different flicker frequencies (presented using a LED) and combinations of spikes and sLFPs: spikes in LGN (n=1332) to sLFPs in LGN, spikes in V1 (n=1028) to sLFP in LGN, and spikes in CA1 (n=72) to sLFP in LGN. Error bars and shadings indicate SEMs.

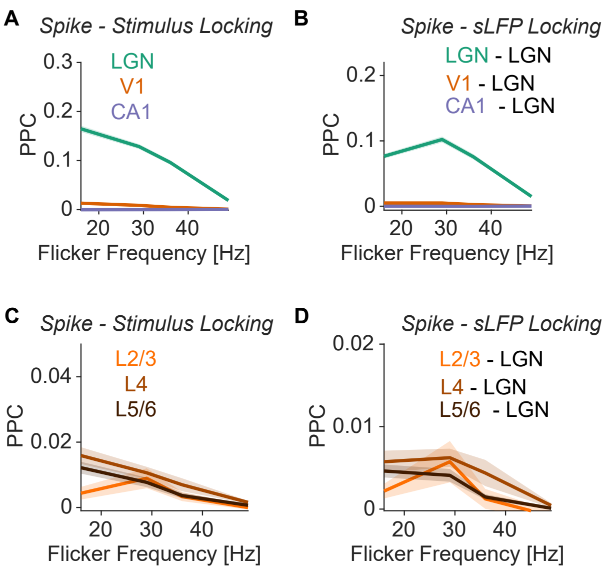

Figure S2: Same as Figure 2 but for monitor stimulation. (A) Spike-stimulus phase locking of neurons in LGN (n=1154), V1 (n=815), and CA1 (n=125) during monitor flicker stimulation. **(B)** Spike-sLFP phase locking for different flicker frequencies (presented using a monitor) and combinations of spikes and sLFPs: spikes in LGN to sLFPs in LGN, spikes in V1 to sLFP in LGN, and spikes in CA1 to sLFP in LGN. Same n as in (A). **(C)** Spike-stimulus phase locking of neurons in different layers of V1 during monitor flicker stimulation ($n_{sup.}$ = 32, $n_{gra.}$ = 279, $n_{inf.}$=504) . **(D)** Spike-sLFP phase locking during monitor flicker presentation between spikes in different V1 layers and the LGN-sLFP. Same n as in (C). Error bars and shadings indicate SEMs.

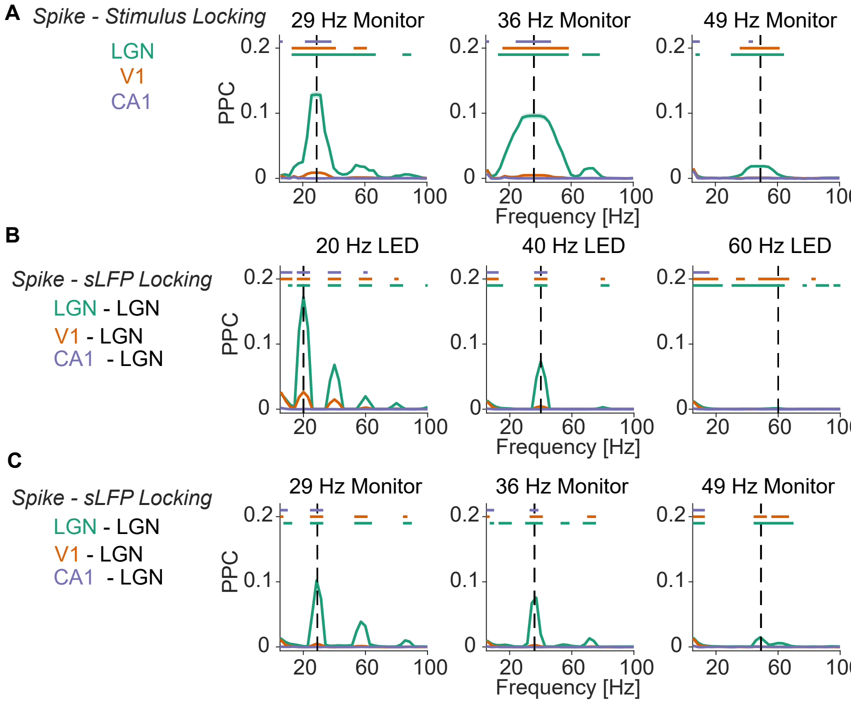

Figure S3: PPC spectra of Neurons in LGN, V1 and CA1, related to Figure 2 and S2. **(A)** Spike-field phase locking during LED flicker stimulation (measured with PPC) for different combinations of spikes and sLFPs: spikes in LGN (n=2386) to sLFPs in LGN (green); spikes in V1 (n=2091) to sLFP in LGN (orange); spikes in CA1 (n=636) to sLFP in LGN (blue). **(B)** Spike-field phase locking during monitor flicker stimulation (measured with PPC) for different combinations of spikes and sLFPs: spikes in LGN (n=1153 to sLFPs in LGN (green); spikes in V1 (n=815) to sLFP in LGN (orange); spikes in CA1 (n=125) to sLFP in LGN (blue). **(C)** Spike-stimulus phase locking during monitor flicker stimulation (measured with PPC) of neurons in LGN (n=1153, green); V1 (n=815, orange) and CA1 (n=125, blue). Randomization test between cells in different areas, FDR correction for multiple comparisons with a threshold of P<0.05, based on 1000 randomizations. Green line: LGN-V1, orange line: LGN-CA1, blue line: V1-CA1. Shadings indicate SEMs.

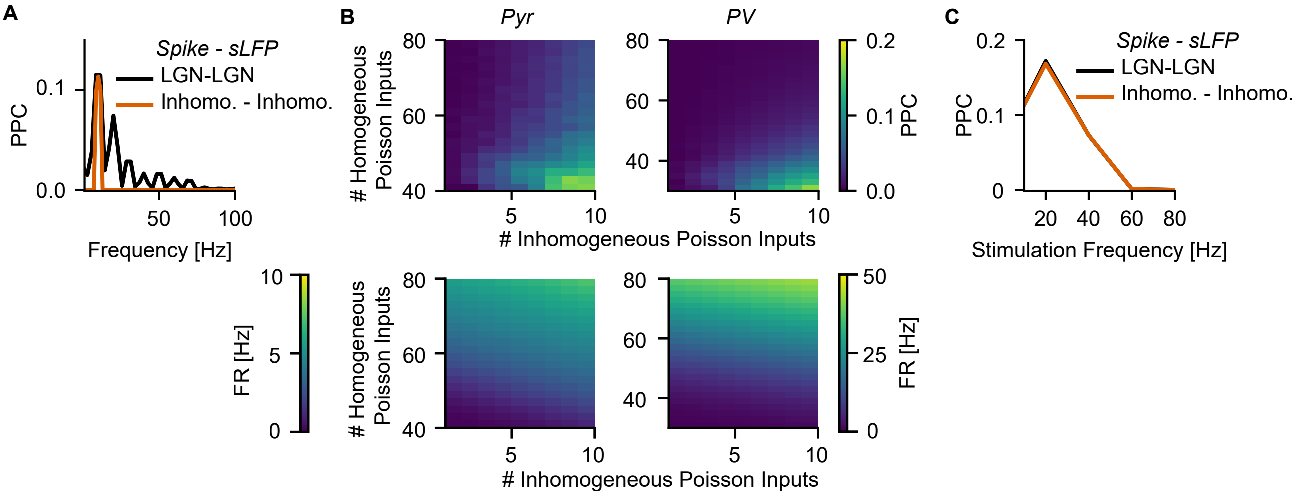

Figure S4: V1 multi-compartmental model during synaptic stimulation, related to Figure 5. **(A)** Spike-sLFP phase locking of LGN neurons during 10 Hz LED flicker stimulation to LGN population activity (black). Phase locking of inhomogeneous Poisson spiking input neurons (orange). Modulation strength of inhomogeneous Poisson spiking input neurons adjusted to reproduce experimentally observed phase locking in LGN. **(B)** Grid scan of the number of homogeneous and inhomogeneous Poisson spiking input spike trains during 10 Hz stimulation. The top plots show the phase locking (PPC) of the pyramidal (left) and PV+ (right) multi-compartmental model to the inhomogeneous Poisson spiking input population. The bottom plots show the firing rates of the corresponding neuron models. The number of homogeneous and inhomogeneous Poisson spiking input trains was optimized to fit the experimental results of BW and NW spiking neurons during 10 Hz LED stimulation. **(C)** Spike-sLFP phase locking (PPC) of single units in LGN to LGN population activity during stimulation at different frequencies (black). The modulation strength of inhomogeneous Poisson spiking input spike trains was adjusted to match experimental observations.

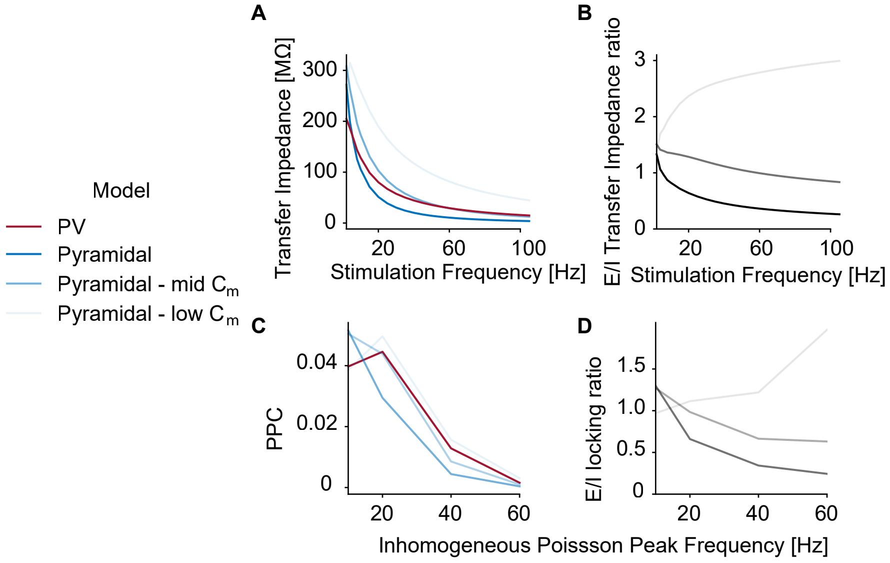

Figure S5: Effects of varying membrane capacitance on dendritic low-pass filtering, related to Figure 5. **(A)** Same simulations as in Figure 5A-D. Transfer impedance of PV+ and pyramidal cell model during sinusoidal current stimulation between 1 and 100 Hz. Increasing the membrane capacitance in pyramidal cell dendrites resulted in a systematic increase in transfer impedance. **(B)** Ratio between the transfer impedance of the pyramidal cell model with scaled membrane capacitance and the PV+ neuron model. **(C)** Same simulations as in Figure 5H-5K. Phase locking of PV+ and pyramidal cell model with scaled membrane capacitance during synaptic stimulation with homogeneous and inhomogeneous spike trains. **(D)** Ratio between phase locking of the pyramidal cell model with scaled dendritic membrane capacitance and the PV+ cell model.
